# Protein-Rich Rafts in Hybrid Polymer/Lipid Giant Unilamellar Vesicles

**DOI:** 10.1021/acs.biomac.3c00972

**Published:** 2024-01-08

**Authors:** Nika Otrin, Lado Otrin, Claudia Bednarz, Toni K. Träger, Farzad Hamdi, Panagiotis L. Kastritis, Ivan Ivanov, Kai Sundmacher

**Affiliations:** † Process Systems Engineering, Max Planck Institute for Dynamics of Complex Technical Systems, Sandtorstrasse 1, 39106 Magdeburg, Germany; ‡ Interdisciplinary Research Center HALOmem and Institute of Biochemistry and Biotechnology, Martin Luther University Halle-Wittenberg, Biozentrum, 06120 Halle/Saale, Germany; § Institute of Chemical Biology, National Hellenic Research Foundation, 11635 Athens, Greece; ∥ Grup de Biotecnologia Molecular i Industrial, Department of Chemical Engineering, Universitat Politècnica de Catalunya, Rambla Sant Nebridi 22, 08222 Terrassa, Spain

## Abstract

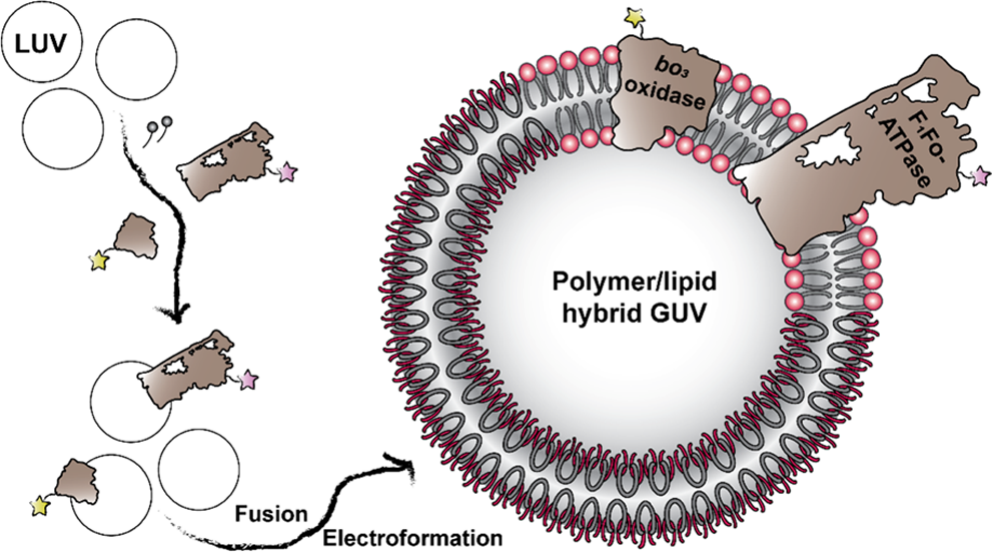

Considerable attention has been dedicated to lipid rafts due to their importance in numerous cell functions such as membrane trafficking, polarization, and signaling. Next to studies in living cells, artificial micrometer-sized vesicles with a minimal set of components are established as a major tool to understand the phase separation dynamics and their intimate interplay with membrane proteins. In parallel, mixtures of phospholipids and certain amphiphilic polymers simultaneously offer an interface for proteins and mimic this segregation behavior, presenting a tangible synthetic alternative for fundamental studies and bottom-up design of cellular mimics. However, the simultaneous insertion of complex and sensitive membrane proteins is experimentally challenging and thus far has been largely limited to natural lipids. Here, we present the co-reconstitution of the proton pump *bo*_3_ oxidase and the proton consumer ATP synthase in hybrid polymer/lipid giant unilamellar vesicles (GUVs) via fusion/electroformation. Variations of the current method allow for tailored reconstitution protocols and control of the vesicle size. In particular, mixing of protein-free and protein-functionalized nanosized vesicles in the electroformation film results in larger GUVs, while separate reconstitution of the respiratory enzymes enables higher ATP synthesis rates. Furthermore, protein labeling provides a synthetic mechanism for phase separation and protein sequestration, mimicking lipid- and protein-mediated domain formation in nature. The latter means opens further possibilities for re-enacting phenomena like supercomplex assembly or symmetry breaking and enriches the toolbox of bottom-up synthetic biology.

## Introduction

Lipid vesicles are one of the fundamental experimental platforms for the integration of membrane proteins (MPs), next to monolayers at the air/water interface, freestanding bilayers (black lipid membranes), and supported monolayers or bilayers. Altogether they enable the biochemical and biophysical characterization of MPs and facilitate the development of MP-targeting drugs. The proteoliposomes are in fact reductionist cell and organelle models, which also grant them a key role in bottom-up synthetic biology.^1^ To assess the functionality of MPs, the latter are predominantly integrated in small (<100 nm) and large (<1 μm) unilamellar vesicles (SUVs and LUVs). However, the easier manipulation and optical access to liposomes in the micrometer range significantly expands the analytical possibilities and allows for studying membrane effects at cellular dimensions and in greater detail. Thus, protein-functionalized giant (>1 μm) unilamellar vesicles (GUVs)^2^ serve as superior model systems from both fundamentally biological and cell-mimicking perspectives.^3,4^

A prominent example of the biological phenomena that are studied in GUVs is lipid rafts. The raft hypothesis proposes that naturally occurring glycosphingolipid- and cholesterol-rich lipid domains are involved in signal transduction, protein sorting, and membrane transport. It has long been believed that cholesterol is an essential component of lipid rafts; therefore, they were traditionally associated with eukaryotic cells. Nevertheless, functional membrane microdomains, homologous in function and organization to the lipid rafts of eukaryotic cells, were recently discovered in bacteria.^5^ Due to their regulatory role in physiological and pathological cellular responses, lipid rafts are often targets of therapeutics such as augmented caveolae-dependent tissue repair.^6^ The hypothesized small (10–200 nm), heterogeneous, and highly dynamic^7^ membrane rafts are easily demonstrated in model membrane systems, where domains can range in size from nanoscale to microscale depending on the lipid mixture.^8^

In the creed of synthetic biology, lipid membranes are also mixed with polymers in order to augment structural and chemical stability^9−12^ as well as chemical diversity,^13^ while retaining biofunctionality and biocompatibility. For instance, embedding the bacterial proton pump ubiquinol *bo*_3_ oxidase in PBd-*b*-PEO/PC^14^ and PDMS-*g*-PEO/PC^12^ LUVs extended its functional lifetime. Furthermore, blending of PC with PDMS-*g*-PEO or with PBd-*b*-PEO preserved the functional shelf life of a light-driven ATP-regenerating module made of bacteriorhodopsin (bR) and ATP synthase to over a month.^15^ Nevertheless, the use of polymer/lipid mixtures as semisynthetic alternatives to the natural protein environment is still sporadic, and the portfolio of MPs is limited to proton pumps, ATPase and SNARE proteins. They were mainly reconstituted into SUVs/LUVs,^14−17^ but in some rare cases also into homogeneous hybrid GUVs;^12,17,18^ nevertheless, their functionality in GUVs was not tested. Yet this new chemistry is a premise for heterogeneity, where the membrane structure is determined by innate parameters like hydrophobic mismatch, polymer/lipid ratio, lipid phase, viscosity and bending rigidity, polymer crystallinity, polymer architecture (e.g., di/triblock or graft), and the method of preparation.^19^ In fact, a relatively small number of amphiphilic copolymers has thus far been explored for the formation of hybrid GUVs.^19^ Heterogeneous polymer/lipid GUVs were prepared with PBd-*b*-PEO,^20−22^ oligo(Asp)-*b*-PPO,^23^ mPEO-*b*-P(MMA-grad-DMAEMA),^24^ mPEO-*b*-PCL,^25^ PDMS-*b*-PEO,^26^ PEO-*b*-PDMS-*b*-PEO,^27^ and PDMS-*g*-PEO.^28,29^ However, MP incorporation and partitioning are yet to be studied in these artificial systems. Pioneering work in this direction was done by Meier and co-authors in 2012, where the mimicry of the membrane with “raft-like” domains was achieved by incorporating OmpF into a lipid/polymer film based on PMOXA-*b*-PDMS-*b*-PMOXA and DPPC,^30^ the latter being in the gel phase, at room temperature. Interestingly, in this system, OmpF was observed to insert into the polymer domains. Furthermore, in their systematic study from 2015, the same group formed monolayers of PDMS-*b*-PMOXA with different PDMS lengths and various phospholipids for direct insertion of MloK1.^31^ There it was found that MloK1 preferentially partitioned in the more fluid phase (copolymer or unsaturated DOPC domains). Note that the native conformation of MloK1 requires a bilayer, and therefore, a denatured protein was used in that study.

Since high proton motive forces and reduced electron transport chains are normally accompanied by oxidative stress,^32,33^ we partially replaced the natural lipid environment by a synthetic alternative to engineer a more robust module for ATP regeneration. To this end, we reconstituted *bo*_3_ oxidase and F_1_F_O_-ATP synthase from *Escherichia coli* in ∼100 nm hybrid LUVs, made of PDMS-*g*-PEO mixed with soy PC.^16^ Along with the above motivation, we aimed to scale the latter system to the micrometer scale, i.e., to form protein-functionalized GUVs, and obtain protein-rich raft-like domains, mimetic to the natural ones. This would enable the use of artificial rafts as a sequestration tool to study MP functions in variable proximity such as signaling, trafficking, etc. In the case of *bo*_3_ oxidase and F_1_F_O_-ATP synthase, bundling together would also shorten the pathway for lateral (membrane-bound) proton transport, potentially resulting in higher ATP synthesis.^34^ While a few strategies for MP reconstitution in GUVs exist, we explicitly sought a method compatible with the sensitive respiratory enzymes to maintain high oxidative phosphorylation activity. There is a common notion that emulsion-based methods like phase transfer^35^ or double emulsions^36^ may be associated with residual oil and surfactants in the membrane, which in turn may negatively affect certain MPs. Even though octanol-assisted liposome assembly was recently found to result in similar membrane fluidity compared to electroformation^37^ and enabled the functional reconstitution of α-hemolysin, we refrained from biphasic approaches. Meanwhile, we screened four other methods for the reconstitution of *bo*_3_ oxidase and F_1_F_O_-ATP synthase: GUV formation in the presence of protein and organic solvent,^38^ in the presence of detergent,^39^ insertion into preformed GUVs via dilution of the detergent,^34^ and fusion/electroformation.^40^ In the latter approach, membrane stacks were formed by partial dehydration of fusing proteoLUV suspensions, followed by rehydration in the presence of an AC electrical field. Fusion/electroformation has been successfully used to reconstitute a number of MPs in lipid GUVs (bR,^40^ Ca^2+^-ATPase,^40^ KvAP,^41^ SNAREs^42^) and even in polymer GUVs (AqpZ, KcsA, and OmpF^43^), whereby the method was adjusted for the specific protein and membrane composition.

In this work, we present the successful adaptation of the above approach for the co-reconstitution of *bo*_3_ oxidase and F_1_F_O_-ATPase into hybrid (PDMS-*g*-PEO/soy PC = 70:30, mol/mol) GUVs. We investigate variations of the method and analyze the GUV size and activity next to the protein distribution. The presented protocol for functional co-reconstitution enables one to study the interactions between functionally coupled MPs in semisynthetic membranes, as well as the bottom-up construction of cell-sized energy-regenerating modules. Finally, by labeling both membrane proteins with fluorescent dyes, we obtained protein-rich lipid rafts.

## Materials and Methods

### Chemicals

Soy PC (95%) and 1,2-dioleoyl-*sn*-glycero-3-phosphoethanolamine-N-(lissamine rhodamine B sulfonyl) (PE-Rho) were purchased from Avanti Polar Lipids. NHS-ATTO 425, NHS-ATTO 514, NHS-ATTO 520, and NHS-ATTO 620 were purchased from ATTO-TEC. PDMS_26_-*g*-(PEO_12_)_2_ was purchased from Dow Corning, which provided a viscosity-average molecular weight of 3000 g mol^–1^, 47% weight fraction of ethylene oxide (2 arms of PEO per PDMS chain on average), and an average degree of polymerization of 12. All other chemicals, including dithiothreitol (DTT) and ubiquinol 1 (Q_1_) were of analytical grade and purchased from Merck.

### Proteins

*E. colibo*_3_ oxidase was expressed from plasmid pETcyo in *E. coli* strain C43 (DE3)(ΔcyoABCDE) and purified as described,^44^ with slight modifications. *E. coli* F_1_F_O_ ATP synthase was expressed from plasmid pBWU13-βHis in *E. coli* strain DK8 (ΔuncBEFHAGDC) and purified as previously described,^45^ with slight modifications. Protein purity analysis was carried out by SDS-PAGE (Figures S1 and S2). *bo*_3_ oxidase was labeled with ATTO 425, ATTO 514, and ATTO 520, and F_1_F_O_-ATPase was labeled with ATTO 620 as described previously.^12^

### (Co-)reconstitution of *bo*_3_ Oxidase and F_1_F_O_-ATPase into LUVs

The (co-)reconstitution protocols for *bo*_3_ oxidase and F_1_F_O_-ATPase into hybrids were slight modifications of our previous protocols.^16^ Briefly, for the reconstitution of *bo*_3_ oxidase, octyl glucoside at the solubilization point (*R*_sol_) was added to hybrids (5 mg mL^–1^ LUVs, final conc. of octyl glucoside 0.11%) for the reconstitution of F_1_F_O_-ATPase, sodium deoxycholate at *R*_sol_ was added to hybrids (5 mg mL^–1^ LUVs, final conc. of sodium deoxycholate 0.065%), and for the co-reconstitution of *bo*_3_ oxidase and F_1_F_O_-ATPase octyl glucoside was added to hybrids (5 mg mL^–1^ LUVs, final conc. of octyl glucoside 0.05%). Next, for individual protein reconstitution, *bo*_3_ oxidase was gently added to hybrids at a final conc. of 0.72, 1.35, or 2.38 μM. Meanwhile, the final conc. of F_1_F_O_-ATPase was 0.68, 0.72, or 2.38 μM. For co-reconstitution, the final conc. of *bo*_3_ oxidase in the reconstitution mixture was 0.72, 0.90, or 2.38 μM and the final conc. of F_1_F_O_-ATPase was 0.45, 0.72, or 2.38 μM. The reconstitution mixture was incubated at 4 °C for 30 min with mild agitation, followed by detergent removal via Bio-Beads SN-2 (Bio-Rad). For the preparation of 200 μL of proteohybrids, the beads were added in 3 subsequent additions, 30 mg of beads each, followed by 30-min incubation at 4 °C and 600 rpm in a thermo shaker. After that, beads were pelleted and the supernatant was collected and stored at 4 °C. If the proteoLUVs were not used for the preparation of proteoGUVs the same day (which was always the case when protein insertion and distribution were analyzed), the vesicle suspension was frozen in liquid N_2_ and aliquots of 20 μL were stored at −80 °C. For measurements of activity of proteins reconstituted in GUVs, proteoLUVs were always used the same day because (1) a large sample volume was required for measurements in a luminometer, and (2) to avoid an increase in activity due to freezing and thawing the samples (see Figure S3).

### Preparation of *bo*_3_-F_1_F_O_-GUVs

Droplets (2 μL) of ∼100 nm proteohybrids (5 mg mL^–1^; containing 0.01 mol % PE-Rho) mixed with 100 nm protein-free hybrids (5 mg mL^–1^; containing 0.01 mol % PE-Rho) in volume ratios of 1:1:2, 1:1:1, and 1:1:0 (for approach I) or 1:2, 1:1, or 1:0 (for approach II) were deposited on ITO-coated glass slides (55 Ω). For analysis of size and protein insertion by fluorescence intensity, *bo*_3_-LUVs (2.38 μM *bo*_3_ oxidase) were mixed with F_1_F_O_-LUVs (2.38 μM F_1_F_O_-ATPase) and protein-free LUVs in a volume ratio of 1:1:1 for approach I, and *bo*_3_-F_1_F_O_-LUVs (2.38 μM *bo*_3_ oxidase and 2.38 μM F_1_F_O_-ATPase) were mixed with protein-free LUVs in a volume ratio 1:2 for approach II. This gave a *bo*_3_:F_1_F_O_:polymer/lipid ratio of 1:1:2700 for both approaches. The proteoLUV film was partially dehydrated for ∼40 min at room temperature (∼22 °C) and ∼20% humidity. Afterward, an electroformation chamber (consisting of two sandwiched ITO-coated glass slides separated by 1.8 mm-thick silicone spacer) was assembled and filled with 1 mM Tris–HCl, pH 7.5, 200 mM sucrose. Electroformation was performed by applying the following sinusoidal electric fields: 50 Hz, 50, 100, 200, 300, 500, 700, and 900 mV for 6 min each; 50 Hz, 1.1 V overnight (∼12 h); and 4 Hz, 2 V for 30 min. For details about the protocol optimization, please see the Supporting Information.

### Monitoring Protein Incorporation and Size Distribution of GUVs

The incorporation of ATTO 425/514/520-labeled *bo*_3_ oxidase and ATTO 620-labeled F_1_F_O_-ATPase in GUVs was analyzed using a Leica STELLARIS 5 confocal laser scanning microscope equipped with an oil immersion 63× (NA 1.4) objective. Commercial software (Leica) LAS X was used for image analysis. Protein distribution was assessed from polyline profiles, which appeared to be highly reproducible (Figure S4). In heterogeneous proteoGUVs, one polyline was drawn through the lipid domain and another through the polymer domain. For statistical evaluation of the size distribution of GUVs, homogeneity and fluorescence intensity of labeled proteins, 60–100 images were taken per sample and the size of 80–220 GUVs was evaluated in LAS X.

### Three-Dimensional (3D) Analysis of ProteoGUVs

ProteoGUVs were immobilized in 0.2 wt % agarose in GUV buffer (1 mM Tris–HCl, pH 7.5, 200 mM sucrose; 218 mOsmol kg^–1^). Shortly, 40 μL of heated agarose solution was deposited on a glass cover slide. On top of the droplet, 20 μL of proteoGUVs was added. After ∼10 min at room temperature, 3D stacks of proteoGUVs were taken using STELLARIS 5. On average 50 stacks were taken for each GUV and 3D images were constructed using LAS X software.

### Monitoring Respiration-Driven ATP Synthesis in ProteoGUVs

Measurements of respiration-driven ATP production were performed by monitoring the luminescence of the luciferin/luciferase assay in bulk vesicle solution. 31.3 μL of *bo*_3_-F_1_F_O_-GUVs were added to 93.8 μL of reaction buffer (20 mM Tris (pH 7.5), 20 mM H_3_PO_4_, 114 mM sucrose; ∼200 mOsmol kg^–1^), and the 1.5 mL Eppendorf tube containing the sample was gently mixed (500 rpm) for 10 min at room temperature to equilibrate pH. Next, 2.26 μL of luciferin/luciferase reagent CLSII and 5.4 μL of 6.96 mM ADP (ultrapure) (final concentration ∼300 μM) was added, and the sample was gently mixed (500 rpm) for another 2 min. Before each measurement, the sample was vortexed in three short bursts, and the baseline was recorded for ∼2 min. As standard, 2.26 μL of 2 μM ATP (final concentration of 36.6 nM) was added and recorded for another ∼2 min. To start the reaction, 1.5 μL of freshly mixed DTT/Q_1_ (6 μL 1 M DTT mixed with 0.25 μL 80 mM Q_1_) was added. When ATP and DTT/Q_1_ were added, the sample was vortexed in three short bursts before continuing the measurement. ATP synthesis was recorded for around 15 min. The ATP production rates were reported as the average of 2–3 replicates, with standard deviation.

### Protein Partitioning into Heterogeneous Hybrid GUVs

Heterogeneous hybrid GUVs (40:59.97:0.03 = PDMS-*g*-PEO/soy PC/PE-Rho, mol %) were formed with conventional electroformation according to ref (16). Due to the low yield and small size (∼5 μm) of GUVs grown in buffer (1 mM Tris (pH 7.5), 200 mM sucrose), we performed a partitioning experiment on GUVs grown in 100 mM sucrose. For spontaneous protein insertion, we added protein in micelles to GUVs in a protein-to-polymer/lipid molar ratio of 1:2700 (the final protein concentration was 0.04 μM). When both *bo*_3_ oxidase-ATTO 425 and F_1_F_O_-ATPase-ATTO 620 were co-reconstituted, the proton pump was added directly after the addition of ATPase. For sedimentation, we deposited 20 μL of GUVs on top of 40 μL of 100 mM glucose on a glass slide and after ∼2 min observed the samples under a confocal microscope (Leica STELLARIS 5).

### Cryo-TEM Analysis of ProteoLUVs

Cryo-TEM was performed on two samples of *bo*_3_-F_1_F_O_-LUVs, whereby one sample contained labeled proteins (*bo*_3_ oxidase-ATTO 425 and F_1_F_O_-ATPase-ATTO 620) and another nonlabeled proteins (*bo*_3_ oxidase and F_1_F_O_-ATPase). For both samples, the *bo*_3_:F_1_F_O_:polymer/lipid molar ratio was 1:1:2700. LUVs composition was PDMS-*g*-PEO/soy PC (70:30, mol %) and they were prepared in 1 mM Tris–HCl (pH 7.5), 200 mM sucrose at 5 mg mL^–1^. The vitrification of the samples and image acquisition was adapted from ref (12). In short, R2/1 type 200 mesh copper Quantifoil holey carbon grids were glow discharged with a PELCO easiGlow (TED PELLA). 3.5 μL of LUV suspension were applied on the glow-discharged grids and vitrified using the Vitrobot Mark IV System (Thermo Fisher Scientific). The sample was back-blotted for 6 s using standard Vitrobot Filter Paper (i.e., Ø55/20 mm grade 595). The grid was then clipped and loaded on a Glacios 200 keV cryotransmission electron microscope (Thermo Fisher Scientific). Movies were acquired on a Falcon 4i direct electron detector using the EPU software package V 3.3.1 (Thermo Fisher Scientific) at a dose of 90 e^–^ Å^–2^ and a pixel size of 0.936 Å. Recorded movies were subsequently corrected for beam-induced motion and drift using RELION^46^ motion correction.^47^ CTF estimation was performed with CTFFIND4,^48^ using the dose-weighted micrographs for the rest of the analysis.

## Results and Discussion

### Formulation of the Protocol for the Preparation of Hybrid ProteoGUVs

Detergent-mediated reconstitution is the most common approach for the reconstitution of MPs of oxidative phosphorylation into LUVs.^16,18,49^ It enables high insertion efficiency and to a large extent control over protein orientation dependent on the type and concentration of detergent. We previously observed that by adding octyl glucoside to preformed hybrid LUVs at the solubilization point (*R*_sol_) we can obtain almost 60% reconstitution efficiency of *bo*_3_ oxidase, which was much higher than the ones obtained with other commonly used detergents for MP reconstitution.^50^ With octyl glucoside at *R*_sol_, around 60% of proton pumps were correctly orientated (pumping in). Meanwhile, sodium deoxycholate at the saturation point (*R*_sat_) and at *R*_sol_ gave the highest reconstitution efficiency for F_1_F_O_-ATPases in comparison to other detergents, whereby *R*_sol_ led to correct orientation of a much higher portion of ATPases.^50^ Unfortunately, such an approach cannot be applied to GUVs due to the complete solubilization of vesicles before their spontaneous reassembly upon detergent removal (newly formed proteovesicles are nanosized). Instead of adding detergent to preformed GUVs, we tried to form hybrid GUVs in the presence of detergent, following the approach previously applied to lipid GUVs.^39^ Electroformation of hybrid GUVs in the presence of detergent (i.e., electroswelling of the polymer/lipid/dodecyl maltoside film) resulted in inefficient GUV formation (Figure S5). A so-called “quick and dirty”, which is rarely used these days, is GUV formation by rehydration under an AC electrical field of a dried film prepared from a solution of lipids and proteins in organic solvent,^38^ which enables simultaneous growth of GUVs and insertion of MPs. Nevertheless, such an approach is typically not compatible with complex MPs, which we also demonstrated here. Electroformation of proteoGUVs from the diethyl ether/polymer/lipid/protein mixture led to the complete loss of protein activity, confirming the detrimental influence of organic solvents on sensitive MPs (see Figure S6 for respiratory-driven ATP synthesis of *bo*_3_-F_1_F_O_-hybrid GUVs and Figure S7 for corresponding micrographs). Furthermore, with both approaches, i.e., GUV formation in the presence of detergent and GUV formation in the presence of protein, the control over the orientation and reconstitution efficiency of MPs is lost. Provided that we had previously secured nearly complete surfactant removal in small vesicles (below the HPLC-MS detection limit of 100 μg L^–1^), next to sustained enzyme activity,^16^ for the scale-up we ultimately resorted to the reported combination of SUV/LUV reconstitution and electroformation, referred to as the fusion/electroformation approach.^40^

### Key Factors in the Preparation of Hybrid ProteoGUVs via Fusion/Electroformation

**Figure 1:**
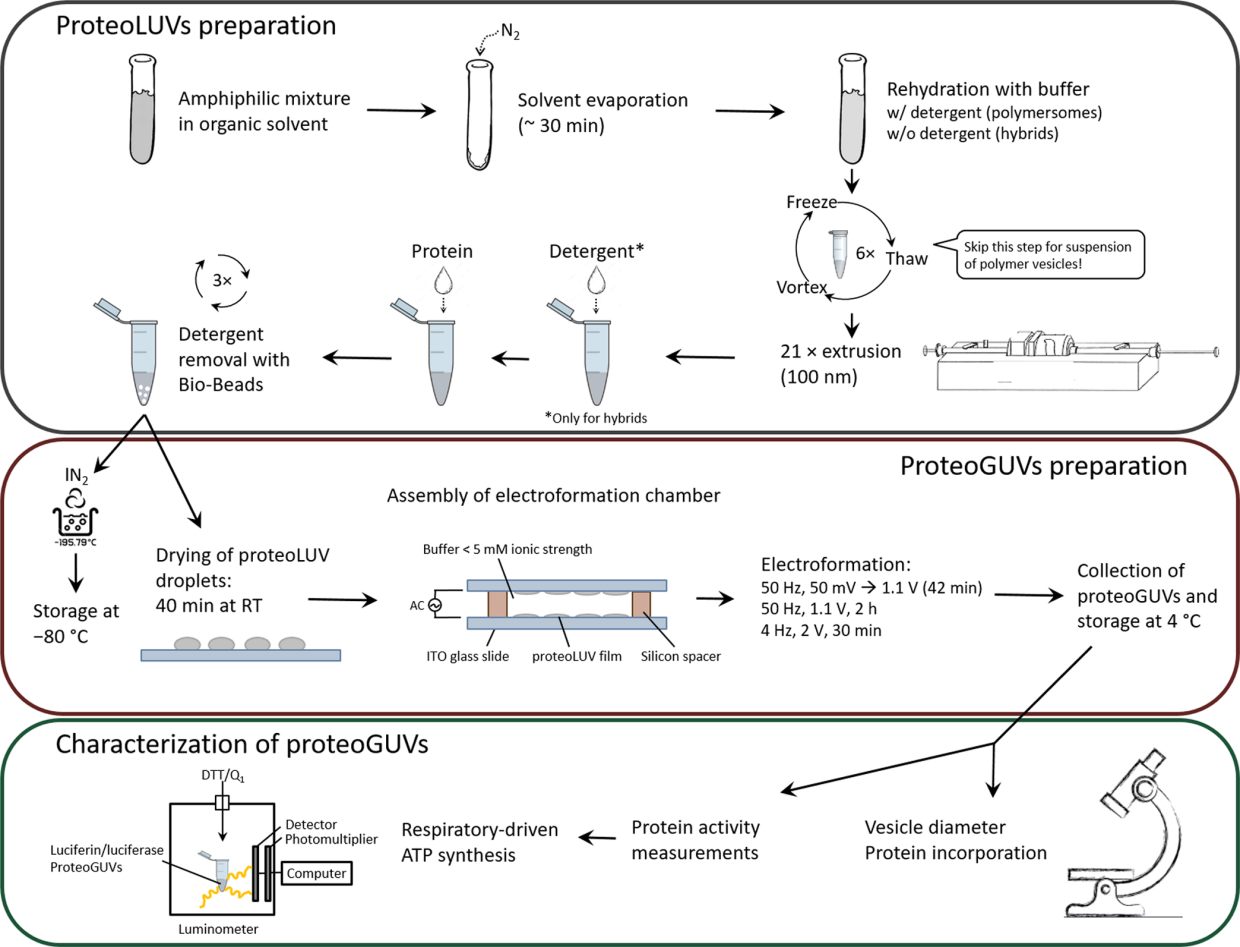
Scheme for the preparation and characterization of the hybrid proteoGUVs. LUVs were prepared by the rehydration of an amphiphilic film and extrusion to unify their size. *bo*_3_ oxidase and F_1_F_O_-ATPase were reconstituted or co-reconstituted in LUVs with the help of detergent. Droplets of proteoLUV suspensions were deposited on ITO-coated glass slides and partially dehydrated, and proteoGUVs were grown by electroformation. The latter were analyzed with respect to size, protein incorporation, and distribution, alongside biological activity.

The proteoGUV formation process involves three successive steps: (1) protein incorporation in LUVs through detergent-mediated reconstitution, (2) partial dehydration of proteoLUVs on ITO-coated glass slides, and (3) hydration under an electric field (Figure 1). The setup for steps 2 and 3 is shown in Figure S8. Overall, the optimization (see the Supporting Information) of the latter approach resulted in >10 μm GUVs and the successful insertion of *bo*_3_ oxidase and F_1_F_O_-ATPase. Starting with LUVs enabled better control over protein orientation and reconstitution efficiency,^50^ while fine-tuning was possible because PDMS-*g*-PEO LUVs could be readily solubilized with a wide concentration range of various detergents. Notably, lower detergent concentrations were required for solubilization of PDMS-*g*-PEO in comparison to PC or block copolymer concentrations, which diminishes the probability of denaturation and the amount of detergent to be removed. After hybrid proteoLUVs were deposited on ITO-coated glass slides, they fused into a thin film after 40 min at room temperature. For efficient dehydration, it was crucial that humidity did not exceed 30% (∼20% was optimal). The process was assessed by analyzing the size distribution after electroformation via dynamic light scattering (DLS); the presence of the starting material (LUVs with a size of approximately 100 nm) indicated poor dehydration and fusion (Figure S9). It should be noted that sufficiently high (>5 mg mL^–1^) concentration of proteoLUVs was necessary for successful electroformation (see the Materials and Methodssection). On the other side, we observed that proteoLUVs deposited at 10 mg mL^–1^ successfully fused but only in part as DLS indicated the significant presence of residual LUVs.

For swelling of the fused hybrid proteoLUV film, we tested some common one-step electroformation protocols used for lipids (see the Supporting Information), which altogether resulted in *bo*_3_-LUVs/GUVs with a diameter of only ∼1 μm (Figure S10). Therefore, the final electroformation protocol combined three steps. The first one was decisive for the yield, whereby we assume that slower initial swelling prevented early LUV film detachment. Meanwhile, the second step, in which membranes continued to swell and grow, determined the final size of GUVs; >2 h were required to obtain 10–40 μm GUVs. By extending the duration to 12 h, GUVs with a diameter of ∼100 μm formed but the majority of those did not detach. For the prolonged protocol, it was crucial that the chamber was moved to 4 °C to retain enzymatic activity. In the third step, GUVs detached at elevated voltage and decreased frequency. Overall, the optimized protocol resulted in GUVs with a median size >10 μm, homogeneous protein distribution (Figures S11 and S12), and the absence of LUVs in the lumen. To the best of our knowledge, this was the first time that polymer/lipid proteoGUVs were formed via fusion/electroformation.

### Bypassing Co-reconstitution Issues in LUVs

**Figure 2:**
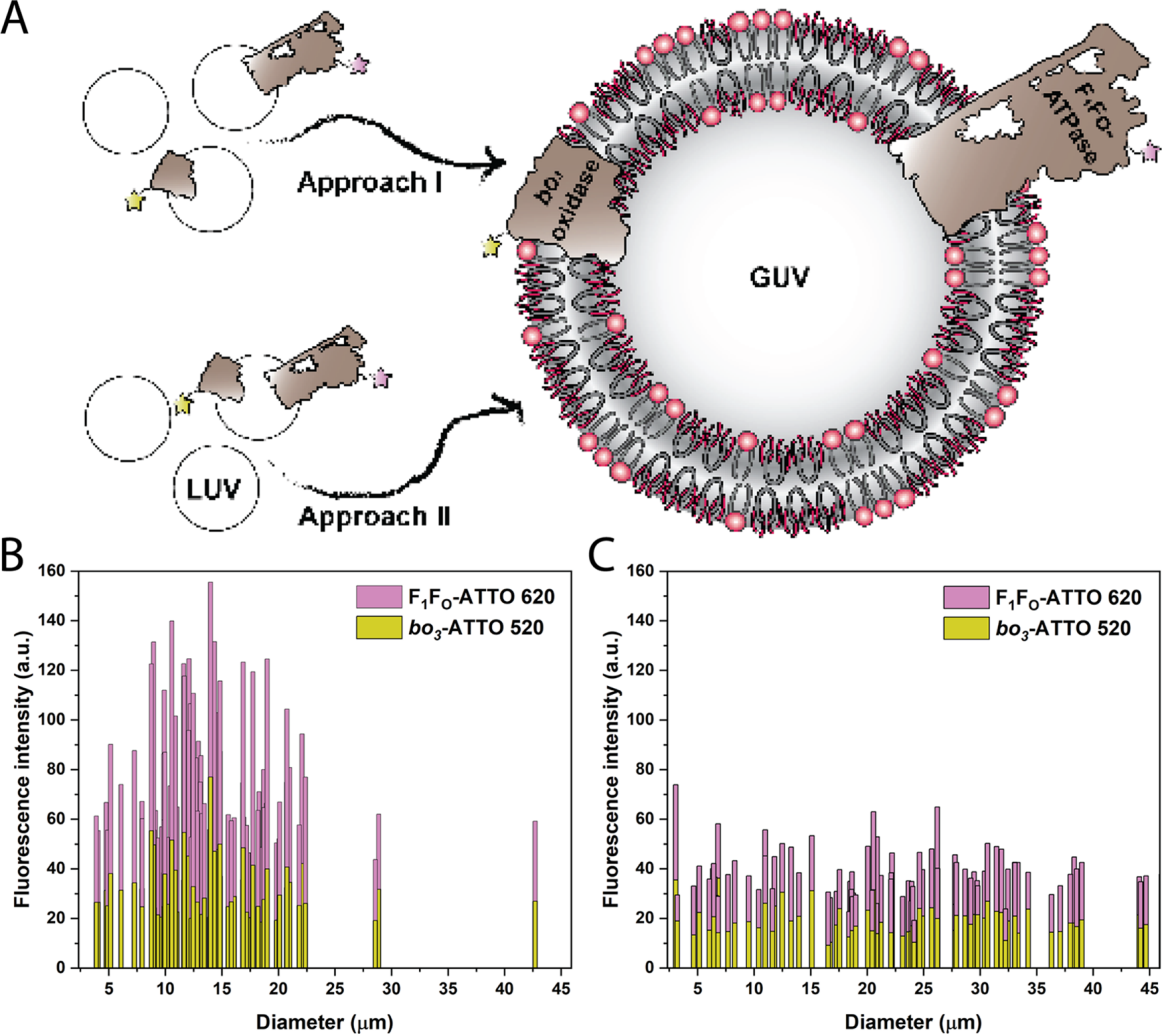
(A) Scheme of the two approaches for co-reconstitution of *bo*_3_ oxidase and F_1_F_O_-ATPase in GUVs. Approach I is based on mixing LUVs with separately reconstituted *bo*_3_ oxidase (*bo*_3_) and ATP synthase (F_1_F_O_), while approach II uses LUVs with co-reconstituted enzymes (*bo*_3_-F_1_F_O_). Fluorescence intensity distribution by the vesicle diameter of hybrid GUVs with co-reconstituted *bo*_3_ oxidase-520 and F_1_F_O_-ATPase-ATTO 620 formed by approach I (B) and approach II (C).

In order to form hybrid GUVs containing two types of MPs we employed two approaches: starting with LUVs with separately reconstituted enzymes (approach I) and with LUVs with co-reconstituted enzymes (approach II) (Figure 2A). Different MPs require different protocols to achieve optimal reconstitution efficiency and orientation, and it is, therefore, often difficult to find the best conditions for simultaneous co-reconstitution. For instance, we previously screened various detergents and concentrations and found that octyl glucoside was optimal for *bo*_3_ oxidase in hybrid LUVs at the solubilization point (*R*_sol_), while sodium deoxycholate gave better results for F_1_F_O_-ATPase.^50^ In this respect, approach I allows for the definition of optimal protocols for individual MPs. Using both approaches, we successfully co-reconstituted the labeled proton pump (*bo*_3_ oxidase-ATTO 520) and proton consumer (F_1_F_O_-ATPase-ATTO 620) and found differences in the degree of protein insertion via confocal microscopy (Figure 2B,C). In hybrid proteoGUVs formed by approach II (at a polymer-to-*bo*_3_ oxidase-to-F_1_F_O_-ATPase molar ratio of 2700:1:1), the average fluorescence intensity of *bo*_3_ oxidase-ATTO 520 was 19 ± 6 au and F_1_F_O_-ATPase-ATTO 620 was 20 ± 5 au (*n* = 87). Meanwhile, approach I led to higher intensities for the proton pump (32 ± 11 au) and for the ATPase (46 ± 17 au) (*n* = 87), while both strategies enabled homogeneous distribution. Note that we compared only the signal of either dye under identical imaging parameters. While approach I led to better protein insertion, the vesicle diameter was on average smaller (14 ± 6 μm for approach I vs 22 ± 11 μm for approach II). The latter derives from the LUV:proteoLUV mixing strategy; in approach I, *bo*_3_-LUVs were mixed with F_1_F_O_-LUVs and protein-free LUVs in a 1:1:1 ratio, meanwhile, in approach II, *bo*_3_-F_1_F_O_-LUVs were mixed with protein-free LUVs in a ratio of 1:2. In general, we observed larger protein-free GUVs in comparison to proteoGUVs under the same growth conditions; therefore, a higher amount of protein-free LUVs (in approach II) seems to facilitate swelling. The reason for such phenomena is that a higher amount of protein forces the vesicles into more areas of forced curvature that mimic the natural curvature demanded by the protein.

### Respiration-Driven ATP Synthesis in Hybrid GUVs

Hybrid polymer/lipid vesicles integrate the advantages of synthetic and natural materials^51^ and may exhibit emergent properties as discussed above. In particular, the integration of *bo*_3_ oxidase in PDMS-*g*-PEO/soy PC LUVs led to higher functional stability and lower proton permeability, compared to both pure polymers and lipids.^12^ Furthermore, the hybrid interface secured near-natural membrane fluidity as a prerequisite for unhindered activity, next to lateral mobility, in line with the diffusion coefficients of smaller MPs in lipid membranes (1.8–10.5 μm^2^ s^–1^). Diffusion coefficients in the hybrid membrane (6.9 ± 1.7 μm^2^ s^–1^ for *bo*_3_ oxidase^12^ and 5.9 ± 0.9 μm^2^ s^–1^ for F_1_F_O_-ATPase^52^) corresponded to the ones observed in DOPC (∼6 μm^2^ s^–1^) upon ATTO labeling.^34^

**Figure 3:**
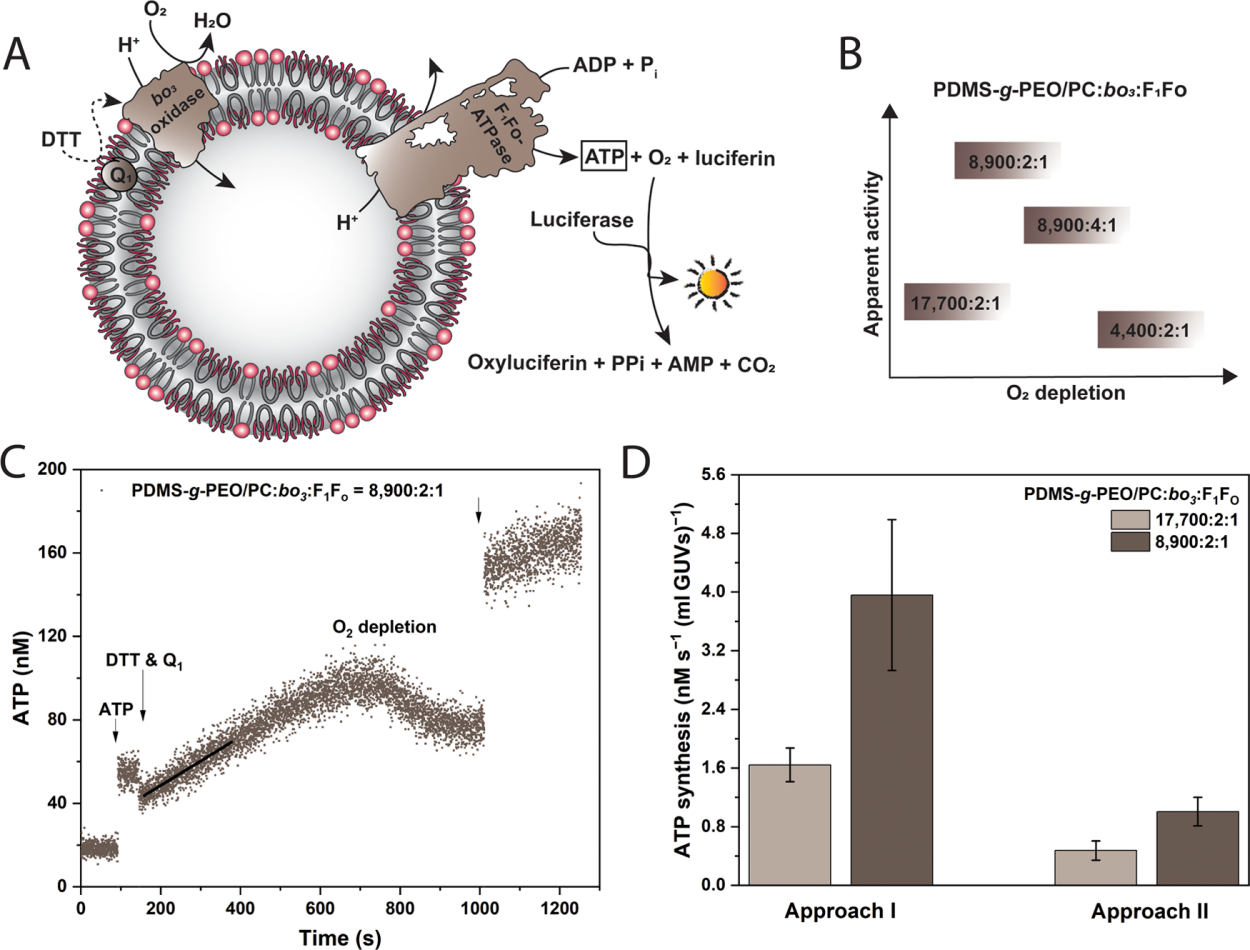
Respiratory-driven ATP Synthesis in hybrid GUVs. (A) Scheme of the functional coupling of enzymes via pH gradient and the ATP detection via luciferin/luciferase. (B) Change in the ATP synthesis rate at four molar ratios of amphiphile-to-*bo*_3_ oxidase-to-F_1_F_O_-ATPase. GUVs were prepared by approach I. (C) Typical ATP measurement in protein-functionalized hybrid GUVs: ATP standard added for internal calibration; proton pumping activated by DTT and Q_1_; and arrows indicate additions and vortexing. (D) Comparison of ATP synthesis rates via approach I and II at two amphiphile-to-protein molar ratios.

The dehydration step at room temperature can have a deleterious effect on MPs.^40^ Although we previously confirmed that this was not the case for *bo*_3_ oxidase,^12^ in this work we tested the activity of F_1_F_O_-ATPase upon its coupling with the proton pump in hybrid GUVs. In parallel, we probed whether the favorable protein orientation in LUVs^16^ (*bo*_3_ oxidase pumping inward and the hydrophilic F_1_ facing outward) was retained after the fusion/electroformation. Thereby, ATP was monitored via the luciferin/luciferase assay (Figure 3A). Hybrid proteoGUVs containing the said respiratory enzymes (nonlabeled) were prepared via approaches I and II and in both cases, and we successfully detected ATP synthesis.

Starting with a molar ratio of mixed amphiphiles to proteins of 8900, we observed an anomalous decrease in ATP production rates upon doubling the overall protein loading, while the ratio (2:1) between *bo*_3_ oxidase and F_1_F_O_-ATPase was kept constant (Figure S13). Furthermore, twice as high a proton pump density (4:1) also led to lower activity. Therefore, we assumed that the unexpected inverse correlation was due to higher oxygen consumption by terminal oxidases. Thus, in the absence of replenishment, oxygen was depleted in the system, which reduced the driving force for the synthesis of ATP and in parallel, might have affected the luciferase assay (Figure 3B). This was also confirmed by the fact that short and mild vortex pulses temporarily restored the rates in all of the tested samples (Figure 3C). In order to decrease the oxygen consumption, we increased the amphiphile proportion to 17,700, which now lowered the activity approximately twice, corresponding to the doubly reduced protein loading (Figure 3D). We note that these rates were normalized to the volume, whereas the exact concentration of the GUV suspensions could not be controlled. Nevertheless, we do not anticipate large variations of the latter, as in all cases we followed the same protocol, while superimposition of the protein and oxygen effects provides a plausible explanation for the peak rate at intermediate enzyme loading. Notably, at identical protein densities higher rates were achieved when proteoGUVs were prepared via approach I (Figure 3D).

### *bo*_3_ Oxidase and F_1_F_O_-ATPase can Sequester Lipids in Domains

**Figure 4:**
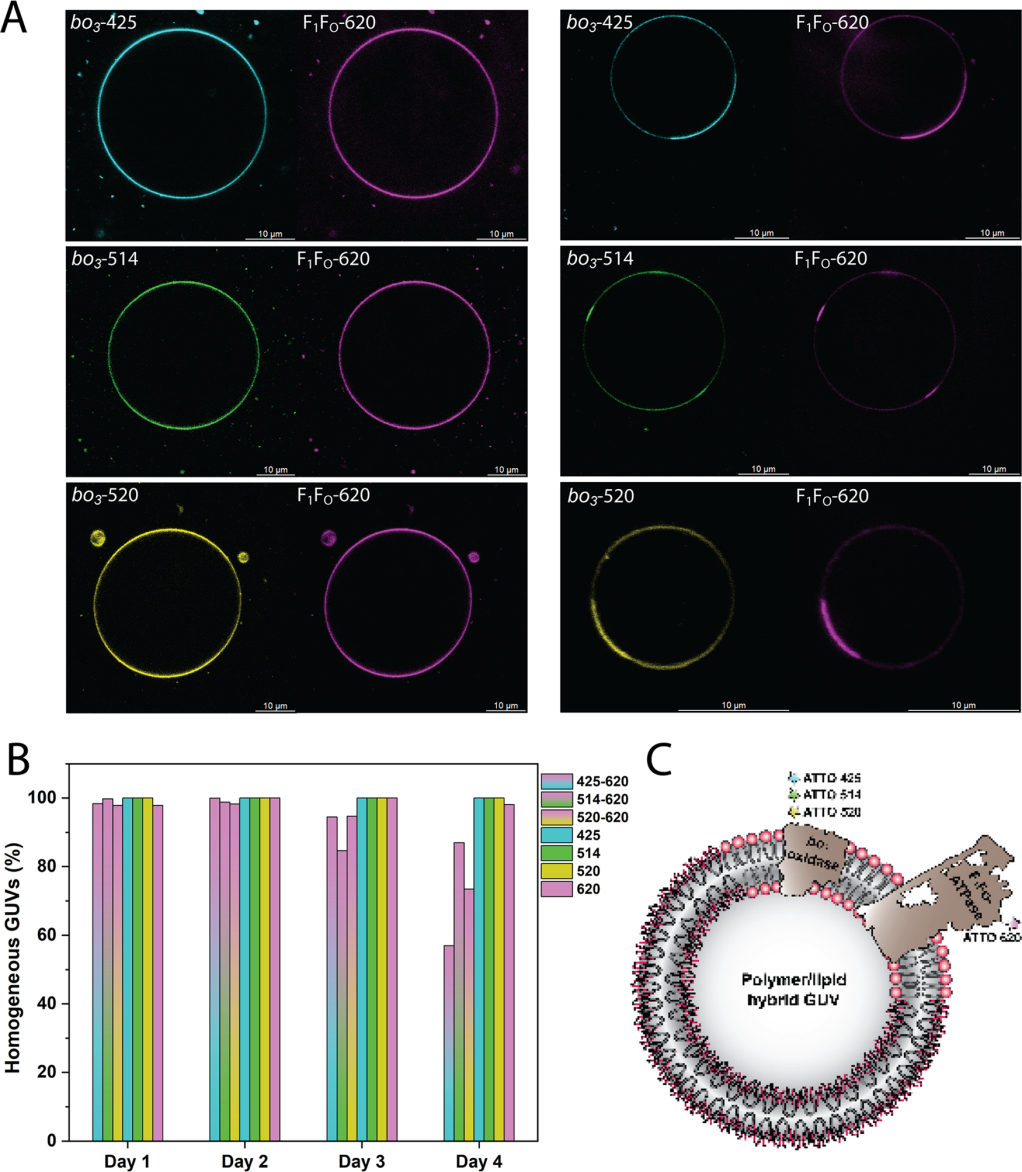
(A) Hybrid GUVs with co-reconstituted *bo*_3_ oxidase and F_1_F_O_-ATPase on day 1 (left) and day 4 (right). *bo*_3_ oxidase was labeled with ATTO 425 (cyan), ATTO 514 (green) or ATTO 520 (yellow), and F_1_F_O_-ATPase was labeled with ATTO 620 (magenta). (B) Portion of homogeneous *bo*_3_-F_1_F_O_-hybrid GUVs, *bo*_3_-hybrid GUVs, and F_1_F_O_-hybrid GUVs over 4 days analyzed from the cross-section. (C) Scheme shows phase separation and formation of functional protein domains. Both proteins are located in the lipid phase.

Directly after formation, the majority of the hybrid proteoGUVs were optically homogeneous, and both enzymes were uniformly distributed (Figure 4A, left panel). However, in a portion of the vesicles we observed separation to lipid and polymer-rich phases over a course of 4 days (Figure 4A, right panel and Figures 4B and S14–S22), whereby *bo*_3_ oxidase and F_1_F_O_-ATPase preferentially partitioned in the lipid domains (Figures 4C and S14–S17). A similar phenomenon was observed with approaches I and II (e.g., see Figure S19). No phase separation was optically detected in protein-free hybrids and neither in proteoGUVs containing individual enzymes (Figure 4B, typical hybrid *bo*_3_-GUVs in Figures S23–S27 and F_1_F_O_-GUVs in Figures S28–S30). Therefore, we probed for the cooperative influence of the proteins and their tags, since ATTO dyes bear different charges, we kept F_1_F_O_-ATPase tagged with ATTO 620 bearing a positive charge. Meanwhile, *bo*_3_ oxidase was tagged with three different ATTO dyes with neutral (ATTO 425), negative (ATTO 514), or positive charge (ATTO 520). Interestingly, we observed the formation of protein-rich lipid rafts for all three cases (Figure 4A, right panel). While F_1_F_O_-ATPase was clearly condensed in the lipid domains, this was not always the case for *bo*_3_ oxidase. When the latter was labeled with ATTO 520, it was present in the polymer phase as well, while higher density was still observed in the lipid phase (Figures S19 and S20), likely due to the repulsive charges of the tags.

**Figure 5:**
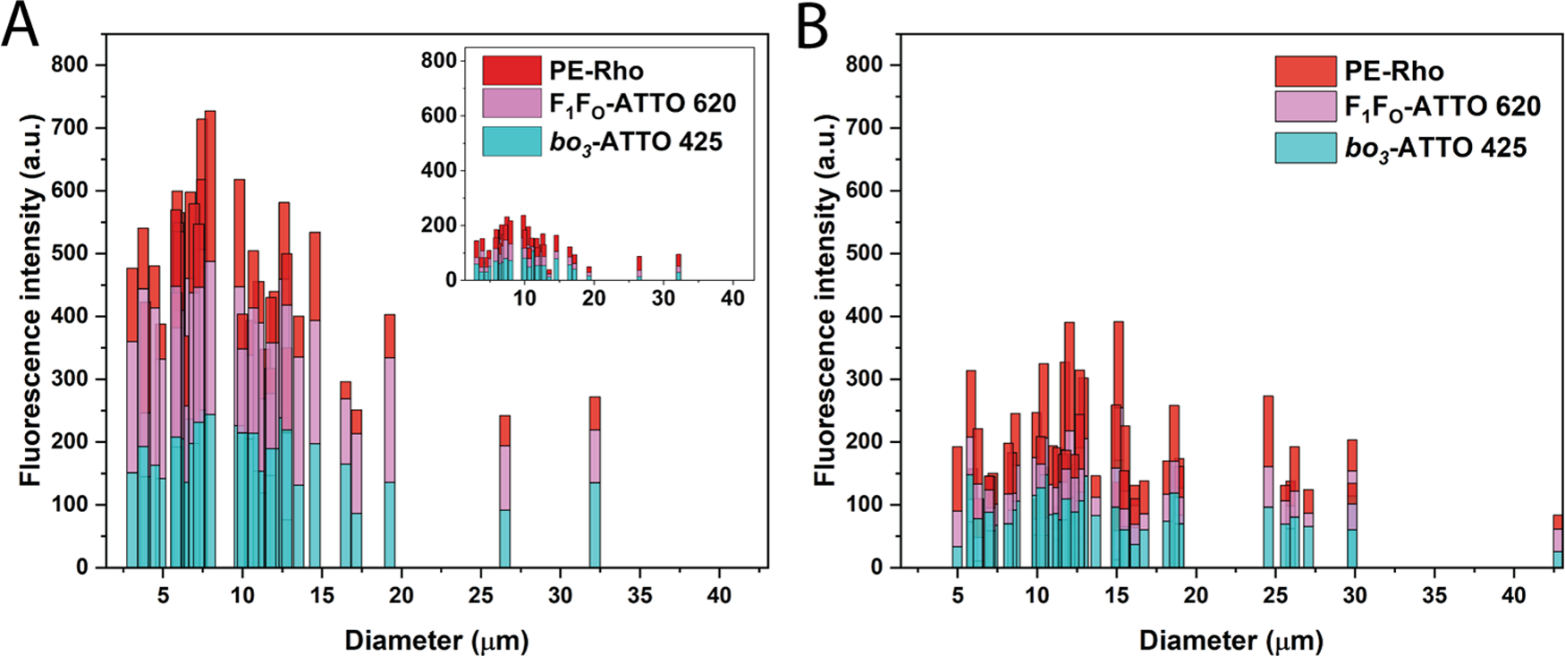
Fluorescence intensity of *bo*_3_ oxidase-ATTO 425, F_1_F_O_-ATPase-ATTO 620, and PE-Rho in (A) heterogeneous and (B) homogeneous hybrid GUVs. For heterogeneous hybrids, fluorescence intensity in protein-rich lipid domains is shown on the main graph; fluorescence intensity in polymer domains is shown in the inset.

We correlated the fluorescence intensity of both proteins with the GUV size in the samples with the most phase separation, i.e., where *bo*_3_ oxidase was labeled with ATTO 425. Interestingly, the majority of heterogeneous GUVs were smaller than the homogeneous ones but occasionally larger (>20 μm) phase-separated GUVs were observed too (Figure 5). As expected, both proteins were enriched in the lipid domains, compared to both the polymer domains and the homogeneous reference, respectively (for *bo*_3_-ATTO 425 181 ± 47 vs 60 ± 26 vs 80 ± 35 au, and for F_1_F_O_-ATTO 620 188 ± 50 vs 37 ± 17 vs 46 ± 17 au, Figure 5). Meanwhile, the analysis of rhodamine intensity confirmed that overall, there was a significantly lower presence of the lipid in the homogeneous GUVs (67 ± 34 vs 103 ± 51 au), which is likely one of the reasons why phase separation did not occur in those hybrids. Protein concentration is another important factor that affects the formation of protein-rich lipid domains. In some cases, higher humidity (>40%) required longer dehydration times (up to 80 min), which led to lower protein concentrations, and these samples rarely exhibited phase separation. Since both the polymer/lipid ratio and protein concentration, play a role in the formation of protein-rich lipid domains, it is likely that decreasing the polymer amount to 60 mol % (minimum amount to still obtain homogeneous GUVs) and increasing protein concentration would lead to faster and more efficient formation of protein-rich lipid domains. However, the presence of a higher amount of polymer is beneficial for system’s chemical stability^12^ and increasing protein concertation leads to a decrease in the GUV size.^52^ The delayed phase separation (over the course of 4 days) in comparison to much faster (several hours) phase separation and budding in protein-free heterogeneous PDMS-*g*-PEO/PC (25:75, mol %) GUVs^28^ is associated with reaching critical lipid domain size to grow further by migrating lipids (and proteins in the current study) and to reach optically detectable size. Since lipid nanodomains are smaller and much rarer in the current system, the process takes longer.

**Figure 6:**
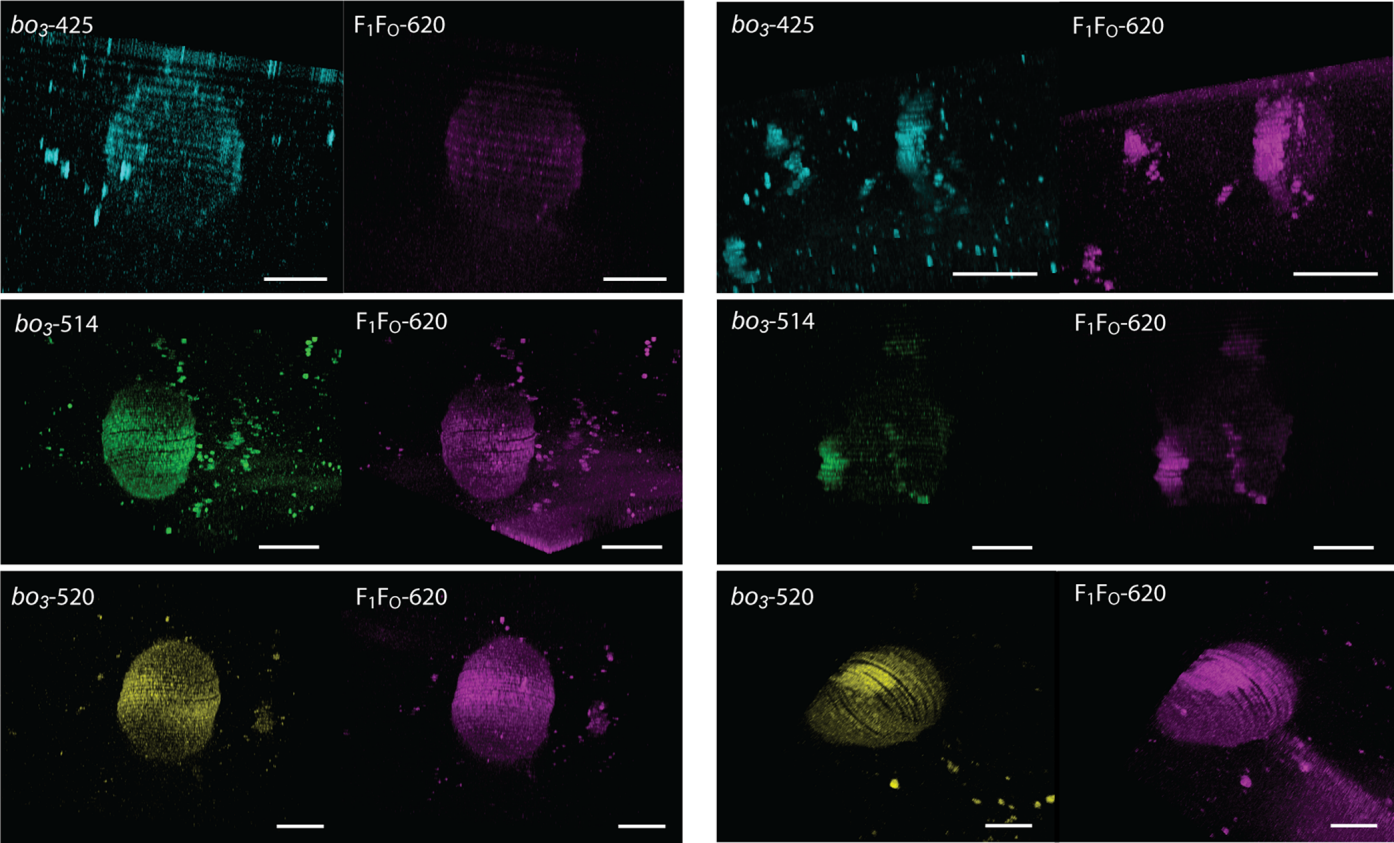
Hybrid GUVs with co-reconstituted *bo*_3_ oxidase labeled with ATTO 425 (cyan), ATTO 514 (green), or ATTO 520 (yellow) and F_1_F_O_-ATPase-ATTO 620 (magenta) on day 1 (left) and day 4 (right). Scale bar: 5 μm.

3D scans revealed that phase separation was more frequent than that observed in the cross-section, and proteoGUVs typically contained 1–3 domains (Figures 6, S17, and S18). Thereby, on day 4 the protein-rich lipid rafts were more pronounced when *bo*_3_ oxidase was tagged with ATTO 425 and 514, and less when tagged with ATTO 520 (Videos S1 and S2).

**Figure 7:**
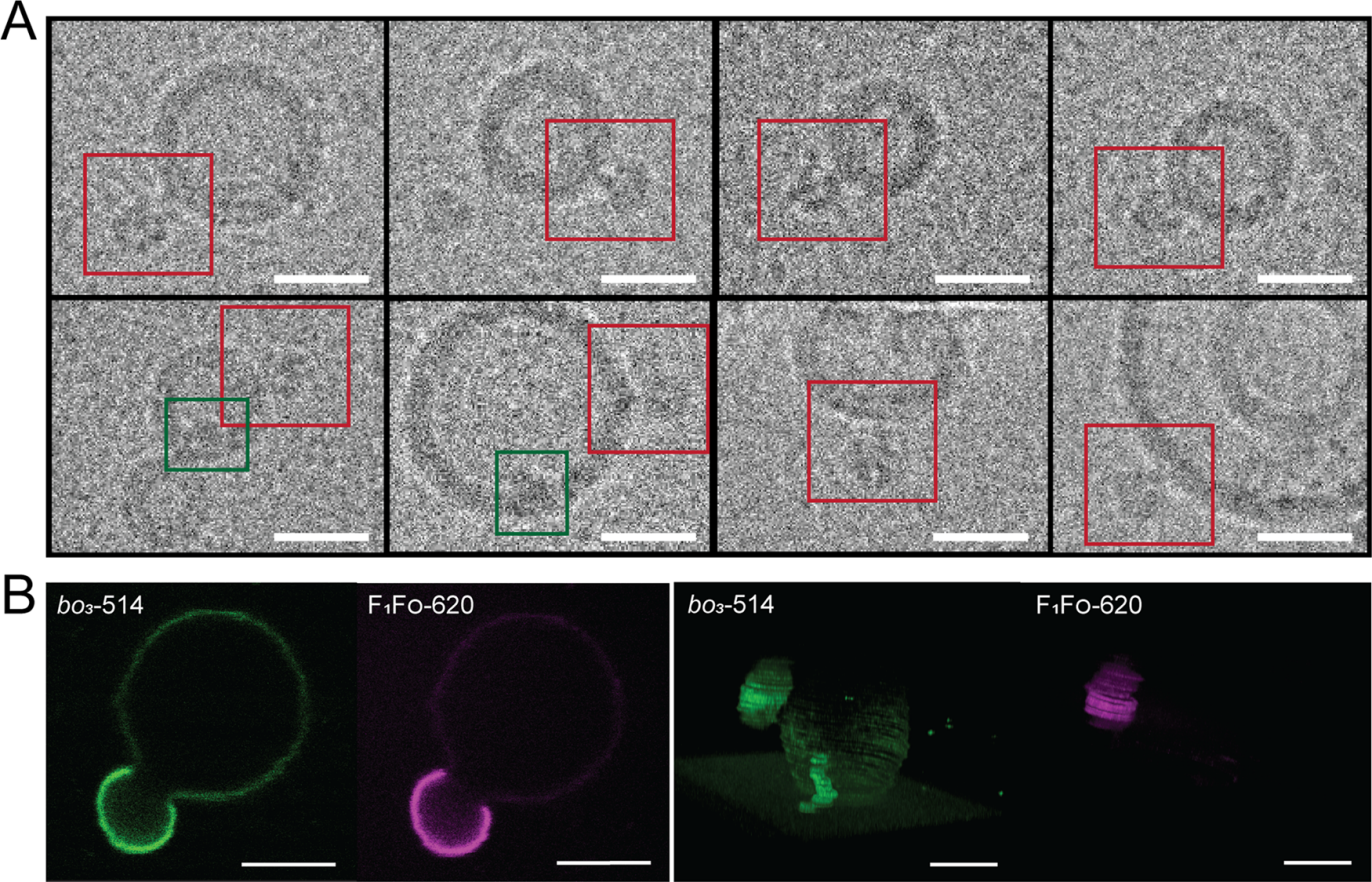
(A) CryoEM images of PDMS-*g*-PEO/soy PC (70:30, mol %) LUVs with co-reconstituted *bo*_3_ oxidase-ATTO 425 and F_1_F_O_-ATPase-ATTO 620. Red boxes indicate F_1_F_O_-ATPase and green ones *bo*_3_ oxidase. Defocus: ∼2 μm (scale bars, 20 nm). (B) Hybrid GUV with co-reconstituted *bo*_3_ oxidase-ATTO 514 (green) and F_1_F_O_-ATPase-ATTO 620 (magenta) undergoing budding (image taken on day 8). Scale bars: 5 μm.

We did not observe phase separation in proteoGUV when proteins were not labeled (Figure S32). Interchangeable use of labeled enzymes (either *bo*_3_ oxidase or F_1_F_O_-ATPase), while the other one was kept native, which resulted in optically homogeneous proteoGUVs only. Therefore, we ascribed the phase separation to the protein dyes because they are known to modify the overall protein charge and the associated interactions, as demonstrated in surface adsorption and cell binding studies.^53,54^ Nevertheless, regardless of the charge of the dye on *bo*_3_ oxidase, we always observed protein-rich lipid domains. Therefore, we believe that next to charge, hydrophobic interactions play a role too. It was previously observed that the contact between less hydrophilic dyes promotes protein–protein interactions. F_1_F_O_-ATPase labeled with less hydrophilic ATTO 647N was shown to interact with *bo*_3_ oxidase labeled with ATTO 594, which was not the case when the former was labeled with more hydrophilic STAR 635 (interactions were deduced via a decrease in lateral diffusion).^34^ In the current case, ATTO 425 and ATTO 520 on *bo*_3_ oxidase are moderately hydrophilic, and so their hydrophobic interactions with ATTO 620 on F_1_F_O_-ATPase are likely promoting protein–protein interactions. Meanwhile, ATTO 514 is highly hydrophilic, which might be the reason we observed less domains. Nevertheless, attractive electrostatic interactions between negative ATTO 514 and positive ATTO 620 still contributed to phase separation. Interestingly, we rarely observed lipid nanodomains in the starting material, i.e., proteoLUVs (Figure 7A) as indicated by the absence of a clear bilayer architecture that we previously detected in soy PC membranes.^12^ The latter was the case regardless of whether proteins were labeled or not. The absence of protein-rich lipid domains on the nanoscale suggested that low membrane curvature (such as that of GUVs) is required for sufficient protein clustering and membrane demixing. Meanwhile, in heterogeneous proteoGUVs, artificial lipid rafts were observed until day 8 and then a portion of the vesicles underwent budding of protein-rich lipid domains (Figure 7B), which corroborates further that the phase separation is a dynamic process.

**Figure 8:**
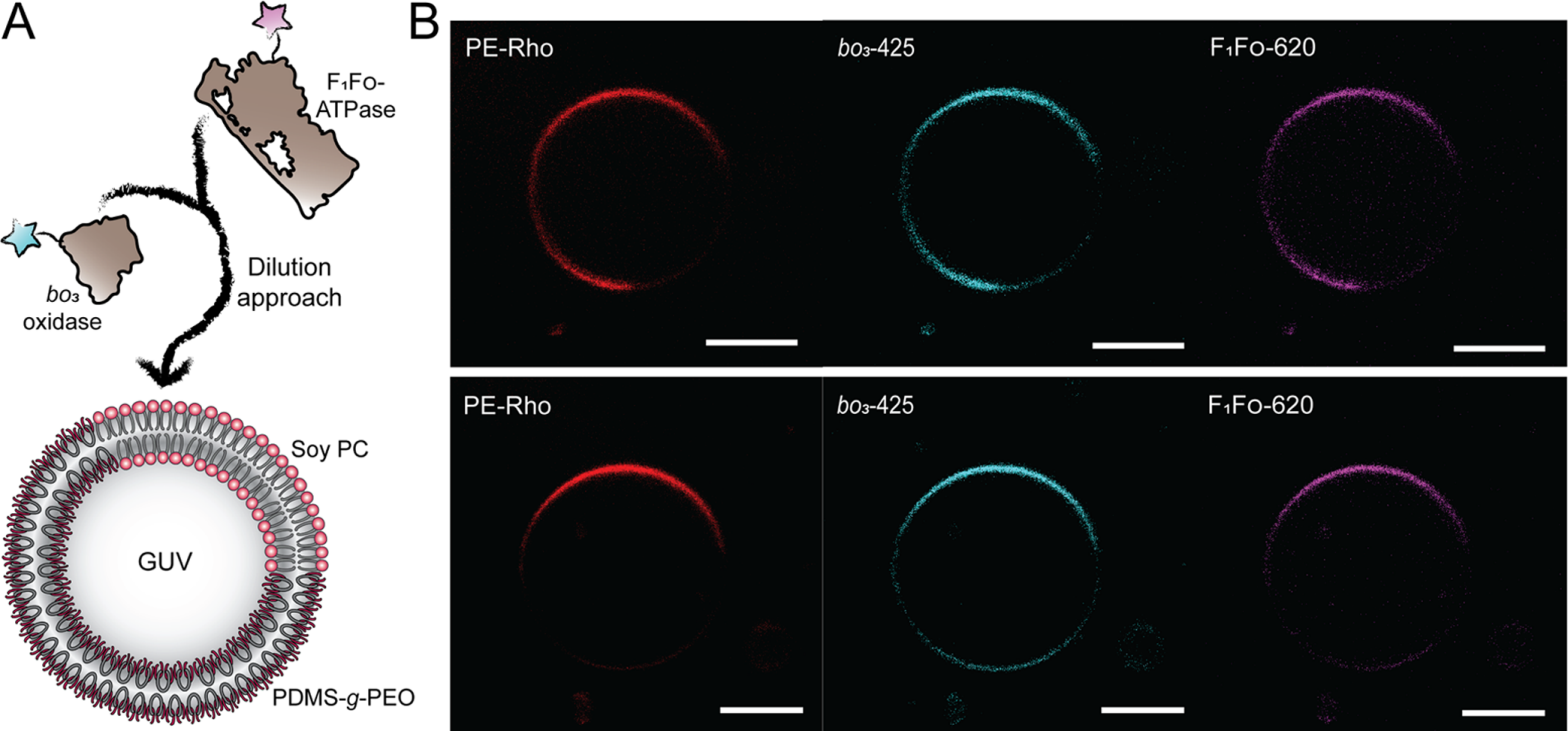
Partitioning of membrane proteins in heterogeneous hybrid GUVs. (A) Scheme showing spontaneous insertion of *bo*_3_ oxidase-ATTO 425 and F_1_F_O_-ATPase-ATTO 620 into the membrane of hybrid GUVs via detergent dilution. (B) Micrographs of two typical proteoGUV where *bo*_3_ oxidase-ATTO 425 (cyan) and F_1_F_O_-ATPase-ATTO 620 (magenta) were inserted exclusively into the lipid domains (red) via a dilution approach. Scale bar: 5 μm.

Although much less pronounced in hybrids than in liposomes, the activity of reconstituted respiratory enzymes drastically decreases with time (even when stored at 4 °C^12^), which conflicts the time scale required for phase separation. Therefore, we prepared GUVs that exhibited lipid rafts right after electroformation by increasing the amount of soy PC (60 instead of 30 mol %). Then we added micellar proteins (*bo*_3_ oxidase-ATTO 425, F_1_F_O_-ATPase-ATTO 620 or both). Due to detergent dilution, the MPs spontaneously inserted into the membrane (Figure 8A). Similar to what was previously observed for MloK1 in PDMS-*b*-PMOXA/DOPC hybrid monolayers,^31^ proteins preferentially distributed to the more fluid phase (11.3 ± 1.5 μm^2^ s^–1^ for soy PC vs 3.6 ± 0.7 μm^2^ s^–1^ for PDMS-*g*-PEO^12^). Along with fluidity, membrane thickness likely plays a role in protein partitioning. Despite the relatively low difference in membrane thickness between soy PC and PDMS-*g*-PEO membrane (4.4 vs 5.3 nm^12^), there is still a hydrophobic mismatch between the hydrophobic part of membrane proteins and the hydrophobic polymer core. PDMS has to compress in the proximity of the proteins in order to overcome the mentioned hydrophobic mismatch.^12^ Such adaptation is not required in the lipid membrane, and therefore proteins tend to partition into an environment more closely resembling the natural one. The mentioned partitioning was observed regardless of whether single or two proteins were used (Figures S33 and 8). Note that micrographs in Figure 8B were taken with different settings than those in Figure 5 (higher laser intensity) because of the lower reconstitution efficiency. Thus, despite being a fast and facile approach to obtain protein-rich lipid rafts, starting with proteoLUVs instead of detergent dilution appears as a more promising method since the concentration of *bo*_3_ oxidase and ATP synthase were 9.3× and 8.8× higher, respectively (Figure S34).

It is not yet clear if in cells, expressed MPs are preferentially inserted into a specific lipid composition or if certain lipids are recruited because of their stronger association with these MPs.^55^ It is conceivable that both processes occur simultaneously and cannot be fully disentangled. We previously observed by fluorescence resonance energy transfer (FRET) that *bo*_3_ oxidase induced local changes in the hybrid membrane composition and sequestered lipids in its vicinity.^12^ Thus, it appears that in some cases, the cumulative hydrophobic and electrostatic attraction between (labeled) proteins, which in turn causes membrane rearrangement and hauls lipids along, suffices to overcome the critical line tension for macroscopic phase separation. Protein-mediated fluid–fluid (liquid ordered/liquid disordered) phase separation was previously observed in lipid vesicles composed of a ternary mixture of sphingomyelin, negatively charged unsaturated lipid dioleoylphosphatidylglycerol (DOPG), and cholesterol.^56^ In the latter system, negatively charged lipids were necessary for the recruitment of Vps32 (ESCRT-III component) at the membrane which triggered domain formation. It should be noted that, in the system presented in the current study, cholesterol is not required to achieve protein-mediated phase separation, and therefore phase separation is not achieved at the expense of the decrease in membrane softness and lateral mobility. Furthermore, both phases (polymer and lipid) are disordered (GP values are −0.52 vs −0.26, respectively^12^), which together with high softness (11.7 κBT for PDMS-*g*-PEO membrane^12^) enables hybrid membranes to efficiently participate in various dynamic processes, such as division and fusion.^17,18^ A related phenomenon in the inverse direction was previously shown for peripheral proteins, whose equivalent charges resided on their side faces, and the resulting repulsion considerably diminished the propensity for demixing.^57^ However, the present system corroborates both scenarios as evidenced by the spontaneous insertion of MPs into preformed domains, providing a tunable system for studying the interplay of factors that lead to functional domains.^58^ Furthermore, the particular superstructural organization of the proton pump and the consumer may be exploited in the future for modulating the length of the proton diffusion pathway (and thus the activity) as protons have been suggested to traverse along the membrane.^59^ Finally, the local accumulation of proteins could mitigate the limited reconstitution efficiency of certain MPs and potentially facilitate the complexation with other respiratory enzymes. However, if the proteins partition into lipid-rich domains that largely exclude the polymer, prolonged activity lifetime, together with membrane stability against ROS, characteristic for homogeneous hybrids,^12^ might be diminished or lost. Meanwhile, the overall proton permeability of heterogeneous protein-functionalized hybrid GUVs would likely be in between the one of protein-free polymersomes and protein-functionalized liposomes, giving overall higher proton permeability than in homogeneous protein-functionalized hybrid GUVs, but still lower than that in protein-functionalized lipid GUVs.^12^

## Conclusions

The stepwise assembly of artificial cells and organelles relies on interfacial functionalization and cytosolic encapsulation. Thereby, the culminating phenomenon of interest (oxidative phosphorylation in the present case) usually requires the combination of a number of different parts, which is experimentally challenging with respect to membranes in particular. In fact, although numerous reconstitutions of individual MPs have enabled their studies, membrane insertion of multiple enzymes has been attempted much less frequently and has been restricted to phospholipids and nanosized vesicles, with only a handful of exceptions. Here, we focused on an integrative approach, which combines the established experiences in protein reconstitution on one side and electroswelling of GUVs on the other. Importantly, mixing LUVs with separately reconstituted MPs enabled higher average ATP synthesis rates on the GUV scale, even though one might anticipate lower activity due to random fusion. Finally, we observed protein-driven phase separation and protein sequestration in lipid domains in otherwise homogeneous polymer/lipid GUVs, which provided an artificial means for the formation of functional protein clusters. The synthetic ability to control sequestration and localization of MPs makes hybrid membranes suitable candidates for applications and studies where specific spatial functionality and proximity are required. Altogether, we believe that the shown integration will facilitate the development of robust biomimicking constructs, while the practical findings may be employed in the vast context of protein co-reconstitution.

## Supporting Information Available

Analysis of purified membrane proteins, additional data on the preparation of LUVs and GUVs, and confocal microscopy images of proteoGUVs. The following files are available free of charge. Supporting Information (PDF) 3D videos of GUVs with protein-rich rafts (avi). The Supporting Information is available free of charge at https://pubs.acs.org/doi/10.1021/acs.biomac.3c00972.

Hybrid GUV with co-reconstituted *bo*_3_ oxidase labeled with ATTO 425 (cyan) and F_1_F_O_-ATPase-ATTO 620 (magenta) on day 4 (MP4)
Hybrid GUV with co-reconstituted *bo*_3_ oxidase labeled with ATTO 520 (yellow) and F_1_F_O_-ATPase-ATTO 620 (magenta) on day 4 (MP4)
Analysis of purified membrane proteins, additional data on the preparation of LUVs and GUVs, confocal microscopy images of proteoGUVs (PDF)

## Abbreviations

PBd-*b*-PEO: poly(butadiene)-*block*-poly(ethylene oxide)
oligo(Asp)-*b*-PPO: oligo(Asp)-*block*-poly(propylene oxide)
mPEO-*b*-P(MMA-grad-DMAEMA): methoxy poly(ethylene oxide)-*block*-P(methacrylates methyl methacrylate-grad-2-(dimethylamino)ethyl methacrylate)
mPEO-*b*-PCL: methoxy poly(ethylene oxide)-*block*-poly(ε-caprolactone)
PEO-*b*-PBD: poly(ethylene oxide)-*block*-polybutadiene
PDMS-*b*-PEO: poly(dimethylsiloxane)-*block*-poly(ethylene oxide)
PEO-*b*-PDMS-*b*-PEO: poly(ethylene oxide)-*block*-poly(dimethylsiloxane)-*block*-poly(ethylene oxide)
PDMS-*g*-PEO: poly(dimethylsiloxane)-*graft*-poly(ethylene oxide)
PMOXA-*b*-PDMS-*b*-PMOXA: poly(2-methyloxazoline)-*block*-poly(dimethylsiloxane)-*block*-poly(2-methyl-oxazoline)
DPPC: 1,2-dipalmitoyl-*sn*-glycero-3-phosphocholine
DPPE: 1,2-dipalmitoyl-*sn*-glycero-3-phosphoethanolamine
DOPC: 1,2-dioleoyl-*sn*-glycero-3-phosphocholine
POPE: 1-palmitoyl-2-oleoyl-*sn*-glycero-3-phosphoethanolamine
CryoEM: cryo-electron microscopy

## References

[ref1] Walde P.; Cosentino K.; Engel H.; Stano P. Giant vesicles: preparations and applications. ChemBioChem 2010, 11 (7), 848–865. 10.1002/cbic.201000010. 20336703

[ref2] Dimova R.; Marques C. The Giant Vesicle Book; CRC Press, 2019.

[ref3] Fenz S. F.; Sengupta K. Giant vesicles as cell models. Integr. Biol. 2012, 4 (9), 982–995. 10.1039/c2ib00188h. 22829218

[ref4] Dimova R. Giant Vesicles and Their Use in Assays for Assessing Membrane Phase State, Curvature, Mechanics, and Electrical Properties. Annu. Rev. Biophys. 2019, 48 (1), 93–119. 10.1146/annurev-biophys-052118-115342. 30811220

[ref5] Bramkamp M.; Lopez D. Exploring the existence of lipid rafts in bacteria. Microbiol. Mol. Biol. Rev. 2015, 79 (1), 81–100. 10.1128/MMBR.00036-14. 25652542 PMC4342107

[ref6] Sviridov D.; Mukhamedova N.; Miller Y. I. Lipid rafts as a therapeutic target: Thematic Review Series: Biology of Lipid Rafts. J. Lipid Res. 2020, 61 (5), 687–695. 10.1194/jlr.TR120000658. 32205411 PMC7193956

[ref7] Pike L. J. Rafts defined: a report on the Keystone symposium on lipid rafts and cell function. J. Lipid Res. 2006, 47 (7), 1597–1598. 10.1194/jlr.E600002-JLR200. 16645198

[ref8] Jacobson K.; Mouritsen O. G.; Anderson R. G. W. Lipid rafts: at a crossroad between cell biology and physics. Nat. Cell Biol. 2007, 9 (1), 7–14. 10.1038/ncb0107-7. 17199125

[ref9] Lee Y.; Chang J.-B.; Kim H. K.; Park T. G. Stability studies of biodegradable polymersomes prepared by emulsion solvent evaporation method. Macromol. Res. 2006, 14 (3), 359–364. 10.1007/BF03219095.

[ref10] Lee J. C.; Bermudez H.; Discher B. M.; Sheehan M. A.; Won Y. Y.; Bates F. S.; Discher D. E. Preparation, stability, and in vitro performance of vesicles made with diblock copolymers. Biotechnol. Bioeng. 2001, 73 (2), 135–145. 10.1002/bit.1045. 11255161

[ref11] Sanson C.; Schatz C.; Le Meins J.-F.; Brûlet A.; Soum A.; Lecommandoux S. Biocompatible and Biodegradable Poly(trimethylene carbonate)-b-Poly(l-glutamic acid) Polymersomes: Size Control and Stability. Langmuir 2010, 26 (4), 2751–2760. 10.1021/la902786t. 19791794

[ref12] Marušič N.; Otrin L.; Zhao Z.; Lira R. B.; Kyrilis F. L.; Hamdi F.; Kastritis P. L.; Vidaković-Koch T.; Ivanov I.; Sundmacher K.; Dimova R. Constructing artificial respiratory chain in polymer compartments: Insights into the interplay between bo_3_ oxidase and the membrane. Proc. Natl. Acad. Sci. U.S.A. 2020, 117 (26), 15006–15017. 10.1073/pnas.1919306117. 32554497 PMC7334566

[ref13] Rideau E.; Dimova R.; Schwille P.; Wurm F. R.; Landfester K. Liposomes and polymersomes: a comparative review towards cell mimicking. Chem. Soc. Rev. 2018, 47, 8572–8610. 10.1039/C8CS00162F. 30177983

[ref14] Khan S.; Li M.; Muench S. P.; Jeuken L. J. C.; Beales P. A. Durable proteo-hybrid vesicles for the extended functional lifetime of membrane proteins in bionanotechnology. Chem. Commun. 2016, 52 (73), 11020–11023. 10.1039/C6CC04207D. PMC5048396 27540604

[ref15] Kleineberg C.; Wölfer C.; Abbasnia A.; Pischel D.; Bednarz C.; Ivanov I.; Heitkamp T.; Börsch M.; Sundmacher K.; Vidaković-Koch T. Light-Driven ATP Regeneration in Diblock/Grafted Hybrid Vesicles. ChemBioChem 2020, 21 (15), 2149–2160. 10.1002/cbic.201900774. 32187828 PMC7496644

[ref16] Otrin L.; Marušič N.; Bednarz C.; Vidaković-Koch T.; Lieberwirth I.; Landfester K.; Sundmacher K. Toward Artificial Mitochondrion: Mimicking Oxidative Phosphorylation in Polymer and Hybrid Membranes. Nano Lett. 2017, 17 (11), 6816–6821. 10.1021/acs.nanolett.7b03093. 29067800

[ref17] Otrin L.; Witkowska A.; Marušič N.; Zhao Z.; Lira R. B.; Kyrilis F. L.; Hamdi F.; Ivanov I.; Lipowsky R.; Kastritis P. L.; Dimova R.; Sundmacher K.; Jahn R.; Vidaković-Koch T. En route to dynamic life processes by SNARE-mediated fusion of polymer and hybrid membranes. Nat. Commun. 2021, 12 (1), 4972 10.1038/s41467-021-25294-z. 34404795 PMC8371082

[ref18] Marušič N.; Otrin L.; Rauchhaus J.; Zhao Z.; Kyrilis F. L.; Hamdi F.; Kastritis P. L.; Dimova R.; Ivanov I.; Sundmacher K. Increased efficiency of charge-mediated fusion in polymer/lipid hybrid membranes. Proc. Natl. Acad. Sci. U. S. A. 2022, 119 (20), e2122468119 10.1073/pnas.2122468119. 35549547 PMC9171793

[ref19] Go Y. K.; Leal C. Polymer–Lipid Hybrid Materials. Chem. Rev. 2021, 121 (22), 13996–14030. 10.1021/acs.chemrev.1c00755. 34752064 PMC11905483

[ref20] Nam J.; Vanderlick T. K.; Beales P. A. Formation and dissolution of phospholipid domains with varying textures in hybrid lipo-polymersomes. Soft Matter 2012, 8 (30), 7982–7988. 10.1039/c2sm25646k.

[ref21] Nam J.; Beales P. A.; Vanderlick T. K. Giant Phospholipid/Block Copolymer Hybrid Vesicles: Mixing Behavior and Domain Formation. Langmuir 2011, 27 (1), 1–6. 10.1021/la103428g. 21133340

[ref22] Khan A. K.; Ho J. C. S.; Roy S.; Liedberg B.; Nallani M. Facile Mixing of Phospholipids Promotes Self-Assembly of Low-Molecular-Weight Biodegradable Block Co-Polymers into Functional Vesicular Architectures. Polymers 2020, 12 (4), 979 10.3390/polym12040979. 32331448 PMC7240622

[ref23] Nishimura T.; Hirose S.; Sasaki Y.; Akiyoshi K. Substrate-Sorting Nanoreactors Based on Permeable Peptide Polymer Vesicles and Hybrid Liposomes with Synthetic Macromolecular Channels. J. Am. Chem. Soc. 2020, 142 (1), 154–161. 10.1021/jacs.9b08598. 31766845

[ref24] Miele Y.; Mingotaud A.-F.; Caruso E.; Malacarne M. C.; Izzo L.; Lonetti B.; Rossi F. Hybrid giant lipid vesicles incorporating a PMMA-based copolymer. Biochim. Biophys. Acta, Gen. Subj. 2021, 1865 (4), 129611 10.1016/j.bbagen.2020.129611. 32272202

[ref25] Go Y. K.; Kambar N.; Leal C. Hybrid Unilamellar Vesicles of Phospholipids and Block Copolymers with Crystalline Domains. Polymers 2020, 12 (6), 1232 10.3390/polym12061232. 32485809 PMC7362021

[ref26] Fauquignon M.; Ibarboure E.; Carlotti S.; Brûlet A.; Schmutz M.; Le Meins J.-F. Large and Giant Unilamellar Vesicle(s) Obtained by Self-Assembly of Poly(dimethylsiloxane)-b-poly(ethylene oxide) Diblock Copolymers, Membrane Properties and Preliminary Investigation of their Ability to Form Hybrid Polymer/Lipid Vesicles. Polymers 2019, 11 (12), 2013 10.3390/polym11122013. 31817266 PMC6960648

[ref27] Dao T. P. T.; Fernandes F.; Ibarboure E.; Ferji K.; Prieto M.; Sandre O.; Le Meins J.-F. Modulation of phase separation at the micron scale and nanoscale in giant polymer/lipid hybrid unilamellar vesicles (GHUVs). Soft Matter 2017, 13 (3), 627–637. 10.1039/C6SM01625A. 27991638

[ref28] Chemin M.; Brun P.-M.; Lecommandoux S.; Sandre O.; Le Meins J.-F. Hybrid polymer/lipid vesicles: fine control of the lipid and polymer distribution in the binary membrane. Soft Matter 2012, 8 (10), 2867 10.1039/c2sm07188f.

[ref29] Chen D.; Santore M. M. Hybrid copolymer–phospholipid vesicles: phase separation resembling mixed phospholipid lamellae, but with mechanical stability and control. Soft Matter 2015, 11 (13), 2617–2626. 10.1039/C4SM02502D. 25687473

[ref30] Thoma J.; Belegrinou S.; Rossbach P.; Grzelakowski M.; Kita-Tokarczyk K.; Meier W. Membrane protein distribution in composite polymer–lipid thin films. Chem. Commun. 2012, 48 (70), 8811–8813. 10.1039/c2cc32851h. 22836593

[ref31] Kowal J.; Wu D.; Mikhalevich V.; Palivan C. G.; Meier W. Hybrid Polymer–Lipid Films as Platforms for Directed Membrane Protein Insertion. Langmuir 2015, 31 (17), 4868–4877. 10.1021/acs.langmuir.5b00388. 25849126

[ref32] Kadenbach B. Intrinsic and extrinsic uncoupling of oxidative phosphorylation. Biochim. Biophys. Acta, Bioenerg. 2003, 1604 (2), 77–94. 10.1016/S0005-2728(03)00027-6. 12765765

[ref33] Murphy M. P. How mitochondria produce reactive oxygen species. Biochem. J. 2009, 417 (1), 1–13. 10.1042/BJ20081386. 19061483 PMC2605959

[ref34] Sjöholm J.; Bergstrand J.; Nilsson T.; Šachl R.; von Ballmoos C.; Widengren J.; Brzezinski P. The lateral distance between a proton pump and ATP synthase determines the ATP-synthesis rate. Sci. Rep. 2017, 7 (1), 2926 10.1038/s41598-017-02836-4. 28592883 PMC5462737

[ref35] Pautot S.; Frisken B. J.; Weitz D. A. Production of Unilamellar Vesicles Using an Inverted Emulsion. Langmuir 2003, 19 (7), 2870–2879. 10.1021/la026100v.

[ref36] Deshpande S.; Caspi Y.; Meijering A. E. C.; Dekker C. Octanol-assisted liposome assembly on chip. Nat. Commun. 2016, 7, 10447 10.1038/ncomms10447. 26794442 PMC4735860

[ref37] Schaich M.; Sobota D.; Sleath H.; Camaa J.; Keyser U. F. Characterization of lipid composition and diffusivity in OLA generated vesicles. Biochim. Biophys. Acta, Biomembr. 2020, 1862 (9), 183359 10.1016/j.bbamem.2020.183359. 32416194 PMC7322398

[ref38] Manneville J. B.; Bassereau P.; Ramaswamy S.; Prost J. Active membrane fluctuations studied by micropipet aspiration. Phys. Rev. E 2001, 64 (2 Pt 1), 021908 10.1103/PhysRevE.64.021908. 11497621

[ref39] Dezi M.; Di Cicco A.; Bassereau P.; Lévy D. Detergent-mediated incorporation of transmembrane proteins in giant unilamellar vesicles with controlled physiological contents. Proc. Natl. Acad. Sci. U.S.A. 2013, 110 (18), 7276–7281. 10.1073/pnas.1303857110. 23589883 PMC3645586

[ref40] Girard P.; Pécréaux J.; Lenoir G.; Falson P.; Rigaud J.-L.; Bassereau P. A new method for the reconstitution of membrane proteins into giant unilamellar vesicles. Biophys. J. 2004, 87 (1), 419–429. 10.1529/biophysj.104.040360. 15240476 PMC1304363

[ref41] Garten M.; Aimon S.; Bassereau P.; Toombes G. E. S. Reconstitution of a transmembrane protein, the voltage-gated ion channel, KvAP, into giant unilamellar vesicles for microscopy and patch clamp studies. J. Visualized Exp. 2015, (95), 52281 10.3791/52281. PMC4354550 25650630

[ref42] Witkowska A.; Jablonski L.; Jahn R. A convenient protocol for generating giant unilamellar vesicles containing SNARE proteins using electroformation. Sci. Rep. 2018, 8 (1), 9422 10.1038/s41598-018-27456-4. 29930377 PMC6013450

[ref43] Itel F.; Najer A.; Palivan C. G.; Meier W. Dynamics of Membrane Proteins within Synthetic Polymer Membranes with Large Hydrophobic Mismatch. Nano Lett. 2015, 15 (6), 3871–3878. 10.1021/acs.nanolett.5b00699. 26013972

[ref44] Frericks H. L.; Zhou D. Z.; Yap L. L.; Gennis R. B.; Rienstra C. M. Magic-angle spinning solid-state NMR of a 144 kDa membrane protein complex: *E. coli* cytochrome bo3 oxidase. J. Biomol. NMR 2006, 36 (1), 55–71. 10.1007/s10858-006-9070-5. 16964530

[ref45] Ishmukhametov R. R.; Galkin M. A.; Vik S. B. Ultrafast purification and reconstitution of His-tagged cysteine-less *Escherichia coli* F1Fo ATP synthase. Biochim. Biophys. Acta, Bioenerg. 2005, 1706 (1–2), 110–116. 10.1016/j.bbabio.2004.09.012. 15620371

[ref46] Zivanov J.; Nakane T.; Forsberg B. O.; Kimanius D.; Hagen W. J. H.; Lindahl E.; Scheres S. H. W. New tools for automated high-resolution cryo-EM structure determination in RELION-3. eLife 2018, 7, e42166 10.7554/eLife.42166. 30412051 PMC6250425

[ref47] Zheng S. Q.; Palovcak E.; Armache J.-P.; Verba K. A.; Cheng Y.; Agard D. A. MotionCor2: anisotropic correction of beam-induced motion for improved cryo-electron microscopy. Nat. Methods 2017, 14 (4), 331–332. 10.1038/nmeth.4193. 28250466 PMC5494038

[ref48] Rohou A.; Grigorieff N. CTFFIND4: Fast and accurate defocus estimation from electron micrographs. J. Struct. Biol. 2015, 192 (2), 216–221. 10.1016/j.jsb.2015.08.008. 26278980 PMC6760662

[ref49] von Ballmoos C.; Biner O.; Nilsson T.; Brzezinski P. Mimicking respiratory phosphorylation using purified enzymes. Biochim. Biophys. Acta, Bioenerg. 2016, 1857 (4), 321–331. 10.1016/j.bbabio.2015.12.007. 26707617

[ref50] Otrin L. Bottom-up Construction of the Artificial Mitochondrion. Ph.D. Thesis, Max Planck Institute for Dynamics of Complex Technical Systems, 2021.

[ref51] Le Meins J. F.; Schatz C.; Lecommandoux S.; Sandre O. Hybrid polymer/lipid vesicles: state of the art and future perspectives. Mater. Today 2013, 16 (10), 397–402. 10.1016/j.mattod.2013.09.002.

[ref52] Otrin N. A Modular Platform for Growth of Hybrid and Polymer Membrane Systems by Vesicle Fusion. Doctoral dissertation, Max Planck Institute for Dynamics of Complex Technical Systems, 2022.

[ref53] Romanowska J.; Kokh D. B.; Wade R. C. When the Label Matters: Adsorption of Labeled and Unlabeled Proteins on Charged Surfaces. Nano Lett. 2015, 15 (11), 7508–7513. 10.1021/acs.nanolett.5b03168. 26491986

[ref54] Yin L.; Wang W.; Wang S.; Zhang F.; Zhang S.; Tao N. How does fluorescent labeling affect the binding kinetics of proteins with intact cells?. Biosens. Bioelectron. 2015, 66, 412–416. 10.1016/j.bios.2014.11.036. 25486538 PMC4836836

[ref55] Shaw A. S. Lipid rafts: now you see them, now you don’t. Nat. Immunol. 2006, 7 (11), 1139–1142. 10.1038/ni1405. 17053798

[ref56] Avalos-Padilla Y.; Georgiev V. N.; Dimova R. ESCRT-III induces phase separation in model membranes prior to budding and causes invagination of the liquid-ordered phase. Biochim. Biophys. Acta, Biomembr. 2021, 1863 (10), 183689 10.1016/j.bbamem.2021.183689. 34224704

[ref57] Mbamala E. C.; Ben-Shaul A.; May S. Domain Formation Induced by the Adsorption of Charged Proteins on Mixed Lipid Membranes. Biophys. J. 2005, 88 (3), 1702–1714. 10.1529/biophysj.104.048132. 15626713 PMC1305227

[ref58] Harder T. Formation of functional cell membrane domains: the interplay of lipid- and protein-mediated interactions. Philos. Trans. R. Soc. London, Ser. B 2003, 358 (1433), 863–868. 10.1098/rstb.2003.1274. 12803918 PMC1693179

[ref59] Nilsson T.; Lundin C. R.; Nordlund G.; Ädelroth P.; von Ballmoos C.; Brzezinski P. Lipid-mediated Protein-protein Interactions Modulate Respiration-driven ATP Synthesis. Sci. Rep. 2016, 6, 24113 10.1038/srep24113. 27063297 PMC4827085

