# Supplementary material for: Protein-Rich Rafts in Hybrid Polymer/Lipid Giant Unilamellar Vesicles
Source: Biomacromolecules. 2024 Jan 8;25(2):778–91. doi: 10.1021/acs.biomac.3c00972 (PMC10865357; doi:10.1021/acs.biomac.3c00972)
Supplement: Supplementary file 3 — bm3c00972_si_003.pdf [file bm3c00972_si_003.pdf]

Supporting Information

Protein-rich rafts in hybrid polymer/lipid giant unilamellar vesicles

Nika Otrin[1], Lado Otrin[1], Claudia Bednarz[1], Toni K. Träger[2], Farzad Hamdi[2], Panagiotis L. Kastritis[2,3], Ivan Ivanov[4,1], Kai Sundmacher[1]*

[1]Process Systems Engineering, Max Planck Institute for Dynamics of Complex Technical Systems, Sandtorstrasse 1, 39106 Magdeburg, Germany

[2]Interdisciplinary Research Center HALOmem and Institute of Biochemistry and Biotechnology, Martin Luther University Halle-Wittenberg, Biozentrum, 06120 Halle/Saale, Germany

[3]Institute of Chemical Biology, National Hellenic Research Foundation, 11635 Athens, Greece

[4]Grup de Biotecnologia Molecular i Industrial, Department of Chemical Engineering, Universitat Politècnica de Catalunya, Rambla Sant Nebridi 22, 08222 Terrassa, Spain

*Corresponding author: nika.otrin@mpi-magdeburg.mpg.de

Contents

Text S1 & Figure S1–S2: Analysis of purified membrane proteins 3

Text S2: Preparation of LUVs 4

Text S3 & Figure S3: Influence of freeze-thaw cycles on the activity of proteoLUVs 5

Figure S4: Analysis of protein insertion 6

Text S4 & Figure S5: Hybrid GUVs grown in the presence of detergent 7

Text S5 & Figure S6–S7: GUV formation by rehydration of a dried film prepared from a solution of polymers, lipids and proteins in organic solvent 7

Text S6: Preparation of proteoGUVs 9

Figure S8–S10: Fusion/electroformation optimization 10

Figure S11–S12: Fusion/electroformation approach I and II 12

Figure S13: Respiratory-driven ATP synthesis 13

Figure S14–S18: Confocal microscopy of *bo*$_3$-ATTO 425-F_1F_O-ATTO 620-hybrid-GUVs 14

Figure S19–S20: Confocal microscopy of *bo*$_3$-ATTO 520-F_1F_O-ATTO 620-hybrid-GUVs 19

Figure S21–S22: Confocal microscopy of *bo*$_3$-ATTO 514-F_1F_O-ATTO 620-hybrid-GUVs 21

Figure S23–S27: Confocal microscopy of *bo*$_3$-ATTO 425/514/520-hybrid-GUVs 23

Figure S28–S30: Confocal microscopy of F_1F_O-ATTO 620-hybrid-GUVs 27

Figure S31: Confocal microscopy of *bo*$_3$-ATTO 514-F_1F_O-ATTO 620-hybrid-GUVs on day 1 29

Figure S32: Confocal microscopy of *bo*$_3$-F_1F_O-hybrid-GUVs 30

Figure S33: Confocal microscopy of protein partitioning in heterogeneous hybrid GUVs 31

Figure S34: Fluorescence intensity of proteins in heterogeneous and homogenous hybrid GUVs 32

Video S1–S2 32

Text S1 & Figure S1–S2: Analysis of purified membrane proteins

SDS-PAGE was carried out according to Schägger and von Jagow (1987) [1] using a 12% acrylamide gel for the F_1F_O-ATPase samples [2] and 16% for the *bo_3* oxidase [3] with a 4% stacking gel each.

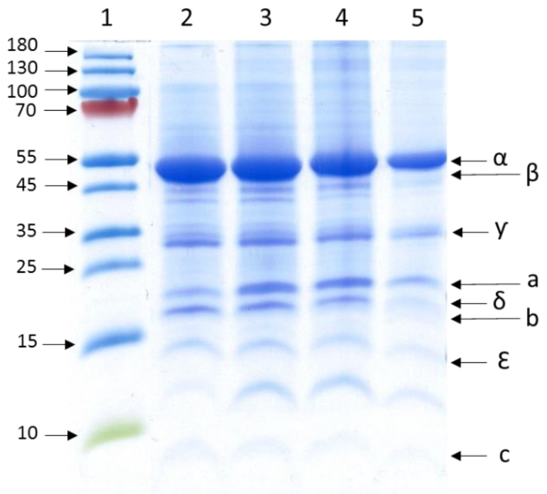

Figure S1. SDS-PAGE analysis of His-tagged F_1F_O-ATPase subunits. Lane 1 shows molecular weight marker (10–180 kDa, Thermo scientific), and lanes 2–5 show subunits of different fractions of F_1F_O-ATPase after His-tag purification.

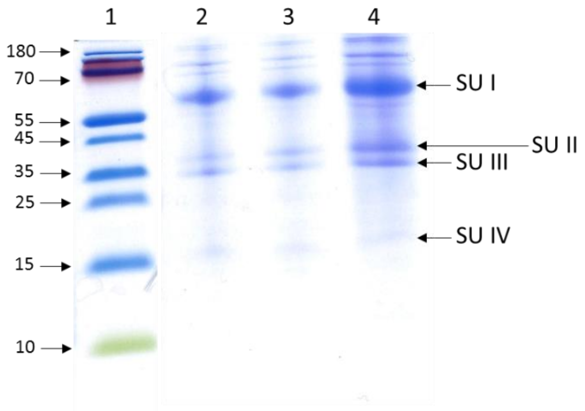

Figure S2. SDS-PAGE analysis of His-tagged *bo_3* oxidase subunits. Lane 1 shows molecular weight marker (10–180 kDa, Thermo scientific), and lanes 2–4 show four subunits of different fractions of *bo_3* oxidase after His-tag purification.

Text S2: Preparation of LUVs

Hybrids were prepared from PDMS-*g*-PEO:soy PC:PE-Rho mixture (70:29.97:0.01, mol%). 5 mg of lipid/polymer mixture in chloroform:MeOH (2:1, v/v) was deposited in a glass vial and the solvent was removed by evaporation under a gentle stream of nitrogen for 30 min. The thin lipid/polymer film was rehydrated with 200 mM sucrose, 1 mM Tris-HCl (pH 7.5) and re-suspended to a final lipid concentration of 5 mg ml^{-1} by vortexing. The suspension of multilamellar vesicles (MLVs) was subjected to 5 freeze-thaw cycles (1 min lN_2, then water bath at 35 °C until thawed completely, followed by 30 s vortexing). Finally, the size and lamellarity of vesicles was unified by extrusion (21 times) through a 100 nm pore (polycarbonate membrane, Mini Extruder).

PDMS-*g*-PEO facilitates LUVs preparation and membrane solubilization

In the first step polymer/lipid mixture in chloroform:MeOH (2:1, v/v) was deposited in glass vials, next solvent was evaporated under gentle N_2 stream (Figure 1) and the thin lipid/polymer film was re-suspended in aqueous media. PDMS-*g*-PEO is liquid at room temperature, leading to facile re-suspension of polymer/lipid film in comparison to lipid (phosphatidylcholine, PC) film (only 2–3 min of vortexing) and much more unlabored than block copolymer film (which requires heating of the media to facilitate the process or mixing for 1–2 days [4]). Second step of LUVs preparation were freeze-thaw cycles, which formed unilamellar vesicles from multilamellar ones. For liposomes and hybrids freeze-thaw cycles were needed to obtain unilamellar vesicles with a single peak observed in dynamic light scattering (DLS) after extrusion, but for polymersomes freeze-thaw cycles could be skipped – polymer prefers to organize in single bilayer than forming multilayers (observed by cryo-TEM [5]). In third step, unilamellar vesicles with various sizes were unified by extrusion. Because of the liquid state of PDMS-*g*-PEO room temperature (22 °C) was sufficient when extruding hybrids or polymersomes (for comparisons, 60 °C is needed for PBD-*b*-PEO [4]). Furthermore, for extruding PDMS-*g*-PEO:PC hybrids lower mechanical force was needed than for liposomes with the same mass concentration. The latter might be due to ~8× lower vesicle concentration (determined by TRPS, tunable resistive pulse sensing). After hybrid film rehydration, hybrids had a maximum diameter of 300 nm (verified by DLS) and were extruded through a membrane with 100 nm sized-pores without difficulty.

While there are three main approaches for insertion of MPs into LUVs, *i.e.* organic solvent-mediated reconstitution, direct incorporation into preformed liposomes and detergent-mediated reconstitution, the last one is commonly used for energy-transducing MP because of its high efficiency and activity retention. The reconstitution is performed in presence of detergent at concentrations of the vesicle saturation point or even higher, *i.e.* above the vesicle to micelle transition [6]. The fluid membranes of liposomes are comparatively easy to solubilize (low detergent concentrations are needed), while the degree of solubilization for reconstitution is determined for the individual MP. On the other hand, in the case of the more rigid membranes of polymersomes, the solubilization requires higher detergent concentrations and stronger detergents (*e.g.* Triton X-100 for PBD-*b*-PEO). For efficient micellization of PBD-*b*-PEO polymer/lipid mixture with sodium cholate 8.6× higher concentrations were used than for reconstitution of bo_3 oxidase in PC and PDMS-*g*-PEO:PC vesicles [4, 7]. In case of octyl glucoside, 16× higher concentration was needed for reconstitution in block copolymer LUVs than for reconstitution of bo_3 oxidase in graft copolymer LUVs [4, 7]. In cases when micellization of block polymer/lipid mixture is possible, the transition to vesicles after detergent removal is difficult to achieve [4]. For hybrids make of PDMS-*g*-PEO graft copolymer various detergents (including mild ones as sodium cholate, which do not damage sensitive proteins) can be used and only very low concentrations are needed, even much lower than for solubilization

of liposomes [7]. This is most likely possible due to the intrinsic surfactant proprieties of PDMS-*g*-PEO [8].

Text S3 & Figure S3: Influence of freeze-thaw cycles on the activity of proteoLUVs

Co-reconstitution of *bo₃* oxidase and F_1F_O-ATPase into LUVs

For proteins co-reconstitution, first, octyl glucoside (final conc. 0.05 %) was added to ~100 nm hybrids (5 mg ml^{-1}) prepared in vesicle buffer (1 mM Tris, pH 7.5, 200 mM sucrose; ~200 mOsmol kg^{-1}), followed by the addition of F_1F_O-ATPase (at final conc. of 0.24 µM) and bo_3 oxidase (at final conc. of 0.48 µM) to obtain polymer/lipid:bo_3 oxidase:F_1F_O-ATPase molar ratio of 8,900:2:1. The reconstitution mixture was incubated at 4 °C, for 30 min, with mild agitation, followed by detergent removal via Bio-Beads SN-2 (Bio-Rad). For preparation of 200 µl of proteohybrids, the beads were added in 3 subsequent additions, 30 mg of beads each, followed by 30 min incubation at 4 °C and 600 rpm in thermo shaker. After that, beads were pelleted and the supernatant was collected. One 20-µl aliquot of proteoLUVs was stored on 4 °C and others were frozen in liquid N_2 and stored at −80 °C.

Monitoring respiratory-driven ATP synthesis in proteoLUVs

Measurements of respiration-driven ATP production were performed via monitoring the luminescence of luciferin/luciferase assay. First, 112.8 µl of reaction buffer (20 mM Tris, pH 7.5, 20 mM KH_2PO_4, 2.5 mM $MgSO_4$, 135 mM sucrose; ~200 mOsmol kg^{-1}), 2.26 µl of luciferin/luciferase reagent CLSII, 3.77 µl of 9.96 mM ADP (ultra-pure) and 2.26 µl of proteoLUVs was mixed in 1.5 ml Eppendorf tube by three short burst of vortexing, and baseline was recorded for ~2 min. As standard, 2.26 µl of 2 µM ATP (final concentration 36.16 nM) was added and recorded for another ~2 min. To start the reaction, 1.5 µl freshly mixed DTT/Q_1 (6 µl 1 M DTT mixed with 0.25 µl 80 mM Q_1) was added. When adding ATP and DTT/Q_1, the sample was vortexed in three short bursts before continuing the measurement. ATP synthesis was recorded for around 15 min. The ATP production rates (Figure S3) were reported as the average of 3 replicates, with standard deviation.

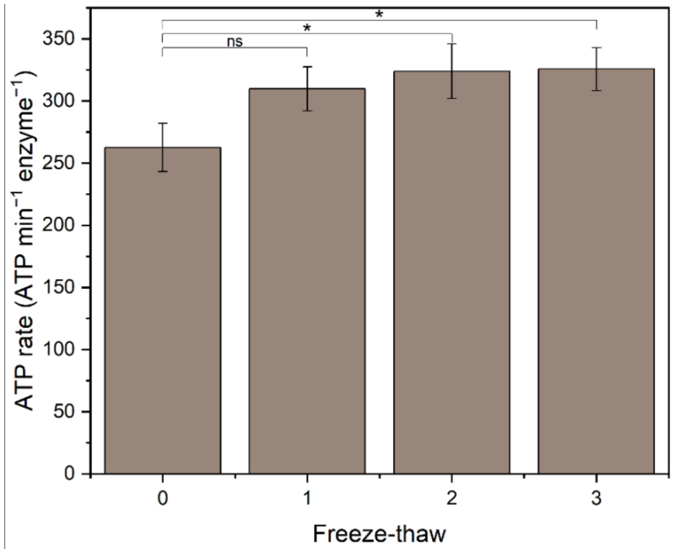

Figure S3. Influence of freeze-thaw cycles on the activity of LUVs with co-reconstituted *bo₃* oxidase and F_1F_O-ATPase, measured via respiratory-driven ATP synthesis. ns, not significant for $P > 0.05$; *$P \leq 0.05$.

Figure S4: Analysis of protein insertion

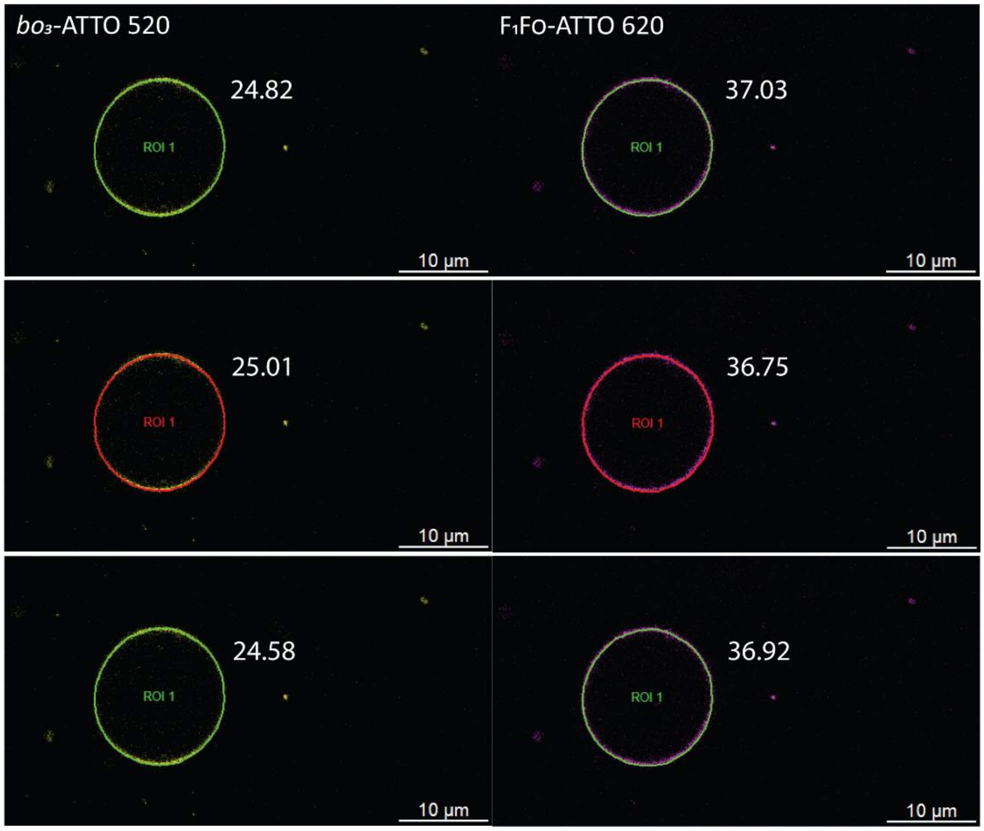

Figure S4. Analysis of protein insertion by drawing polyline. Three panels show the same GUV with co-reconstituted *bo₃* oxidase-ATTO 520 and F_1F_O-ATPase-ATTO 620 analyzed three times with polyline profile and the mean values of fluorescence intensity (a.u.) for each protein are noted.

Text S4 & Figure S5: Hybrid GUVs grown in the presence of detergent

Preparation of DDM-GUVs

First, 1 mg of PDMS-*g*-PEO:soy PS:PE-Rho mixture (70:29.95:0.05 mol%) containing 1.927 mM DDM was dissolved in 1 ml of 2:1 chloroform:methanol (v/v). 15 µl of those solutions were spread on each of both glass slides of a homemade electroformation device composed of two electrodes (glass slides coated with indium tin oxide with resistivity of 55 Ω) and a silicone spacer (1.81 mm thick). Solvent was evaporated under a gentle stream of nitrogen and the electroformation chamber was filled with 1 mM Tris (pH 7.5), 200 mM sucrose. A sine wave at 2 V, 10 Hz, was applied for 60 min, followed by detachment step with square wave at 1 V, 2 Hz, for 15 min. The final concentration of DDM in the sample was 0.2 mM.

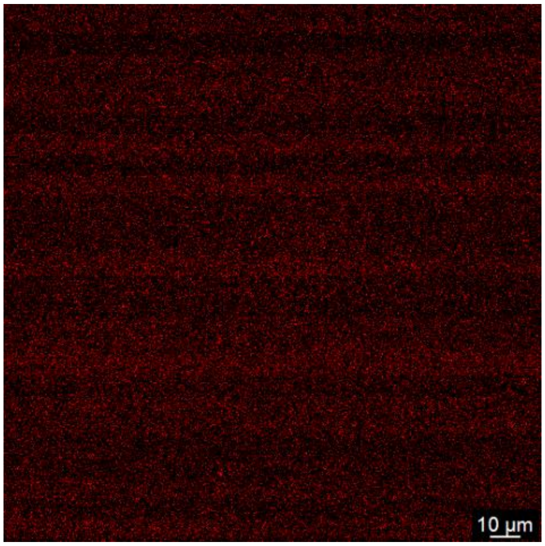

Figure S5. Representative micrograph of PDMS-*g*-PEO/soy PC/PE-Rho/DDM sample after electroformation. As evident, no GUVs could be found.

Text S5 & Figure S6–S7: GUV formation by rehydration of a dried film prepared from a solution of polymers, lipids and proteins in organic solvent

Preparation of proteo-GUVs

The 0.5 mg of PDMS-*g*-PEO:soy PS:PE-Rho mixture (70:29.95:0.05 mol%) were dissolved in 500 or 495.5 µl of diethyl ether, for the control and for preparation of protein-functionalized GUVs, respectively. Next, *bo₃* oxidase and F_1F_O-ATPase were added at the final concentration of 96.3 and 48.2 nM, respectively (to obtain polymer/lipid-to-*bo₃* oxidase-to-F_1F_O-ATPase molar ratio of 8,900:2:1), and the mixture was vortexed for 30 sec. Next, 15 µl of those mixtures were spread on each of both glass slides of a homemade electroformation device composed of two electrodes (glass slides coated with indium tin oxide with resistivity of 55 Ω) and a silicone spacer (1.81 mm thick). Solvent was evaporated under a gentle stream of nitrogen and the electroformation chamber was filled with 1 mM Tris (pH 7.5), 200 mM sucrose. A sine wave at 2 V, 10 Hz, was applied for 60 min, followed by detachment step with square wave at 1 V, 2 Hz, for 15 min.

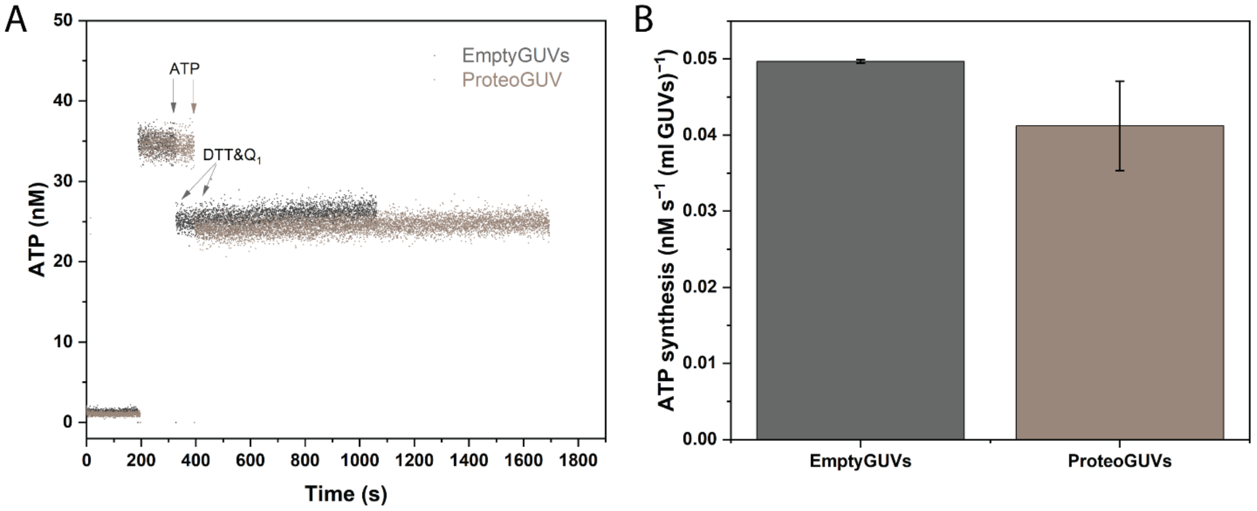

Figure S6. Respiratory-driven ATP synthesis in hybrid GUVs formed by rehydration under an AC electrical field of a dried film prepared from a solution of polymers, lipids and proteins in diethyl ether. A) An example of ATP measurement in empty and protein-functionalized GUVs: ATP standard added for internal calibration; proton pumping activated by DTT and Q_1; arrows indicate additions and vortexing. B) Comparison of ATP synthesis rates in empty and protein-functionalized GUVs.

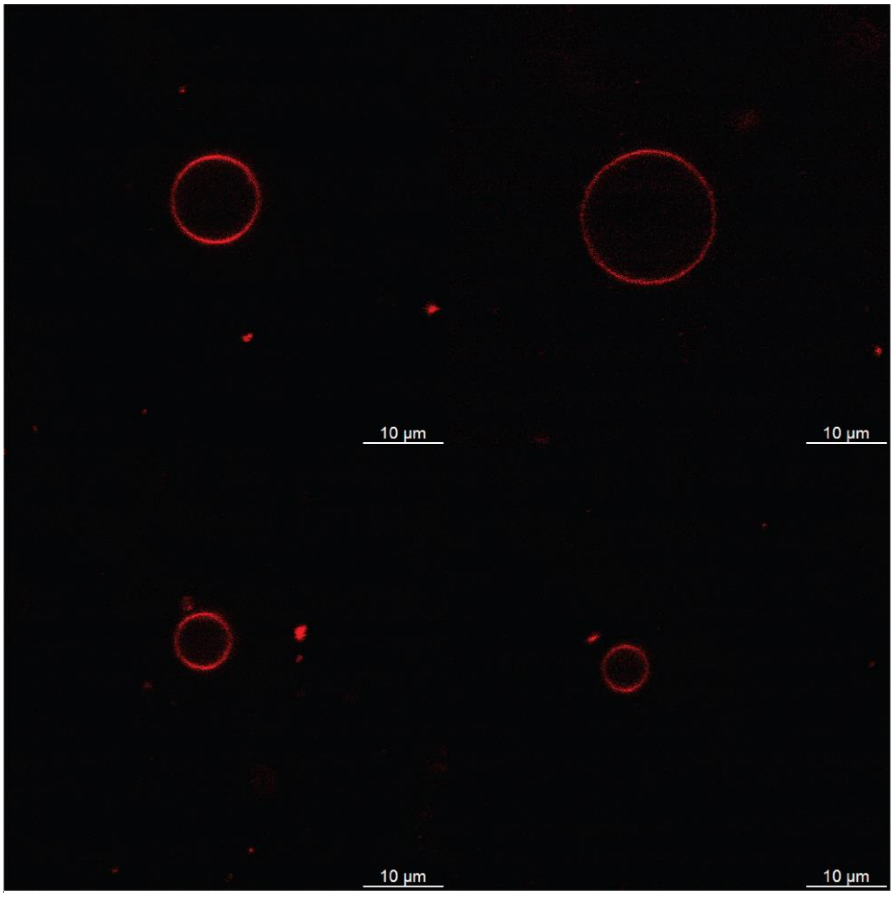

Figure S7. Hybrid GUVs with co-reconstituted *bo*$_3$ oxidase and F_1F_O-ATPase used for measurement of ATP synthesis. GUVs were formed by rehydration under an AC electrical field of a dried film prepared from a solution of polymers, lipids and proteins in diethyl ether. Membrane was tagged with 0.05 mol% PE-Rho (red).

Text S6: Preparation of proteoGUVs

Preparation of *bo₃*-F₁F₀-GUVs

The initial fusion/electroformation experiments were performed with protein-free LUVs. Once the protocol to obtain high yield of GUVs with appropriate size (> 10 µm) was established, proteoGUVs were prepared from proteoLUVs. Varying the dehydration and electroformation steps allowed to obtain *quality* hybrid GUVs (with diameter of 10–30 µm, high yield, without intravesicular structures, and high protein loading).

Dehydration conditions. For successful fusion of LUV membranes and formation of >20 µm hybrid GUVs, it was crucial to deposit on ITO-coated glass slides a layer of LUVs with optimal thickness. Various vesicle concentrations (0.1–10 mg ml^{-1}) and different deposition procedures were tested (spreading 2–20 µl of vesicle suspension on plasma cleaned ITO slides or depositing 0.2–2 µl droplets of vesicle suspension). Deposition of the LUV suspension in droplets was more efficient for vesicle fusion (the DLS peak intensity at 100 nm decreased substantially or even disappeared). Large droplets of relatively highly concentrated vesicles had to be deposited; this can be explained by the lower amount (~8×) of hybrid particles (*i.e.* LUVs) shown by TRPS, compared to the lipid ones for the same mass concentration. Spreading the droplets or depositing smaller (<1 µl) droplets decreased the fusion efficiency (large amount of LUVs was left in the samples after electroformation). Various dehydration procedures were tested: 2 h and overnight in desiccator (at room temperature and 4 °C) in presence and absence of saturated NaCl [9], and 30–60 min at room temperature. The water removal from hybrid LUVs films was facilitated in comparison to lipid LUV film. This is most likely associated to the different concentration of deposited vesicles. The hybrid LUV film behaved similarly to the polymer film because of the high molar percent of polymer in hybrids (70 mol%).

Electroformation protocol. Various electroformation protocols were tested aiming to obtain a high yield of >10 µm GUVs. With most commonly used protocols for preparation of lipid GUVs in low (10 Hz, 1.1 V, 1–3 h) and high salt buffers (500 Hz, 1.1 V, 1–3 h), hybrid *bo₃*-GUVs were formed with maximum diameter of around 1 µm. With the protocol that we previously used for preparation of hybrid GUVs (conventional electroformation from lipid/polymer film, *i.e.* sine wave for 40 min (2 V, 10 Hz), followed by square wave for 15 min (1 V, 2 Hz) [7]), larger (>5 µm) protein-free GUVs were formed, but the size of *bo₃*-GUVs was still around 1 µm. Assuming the need of slower initial swelling to prevent the early LUVs film detachment, we applied a protocol consisting of three subsequent steps: first, the voltage was slowly increased for 42 min (starting with 50 mV and increasing in 6 min-steps to 1.1 V), in which the fused membranes film started to swell. Second, swelling and growing continued at constant voltage and third, the GUVs detached at an elevated voltage and decreased frequency. Comparison of hybrid GUVs prepared with the two different electroformation protocols is shown in Figure S5. Extending the second part from 2 h to overnight (ca. 12 h) led to increased yield of 20–30 µm GUVs. For the most experiments, the yield was high enough with shorter second swelling part, and to retain protein activity (which was decreasing faster at room temperature), the shorter, 2 h swelling step was preferred. For the overnight protocol, during the first electroformation step chamber was at room temperature, while for next two steps chamber was transferred to an ice box (to avoid moisture contact, chamber was protected with plastic bag).

Figure S8–S10: Fusion/electroformation optimization

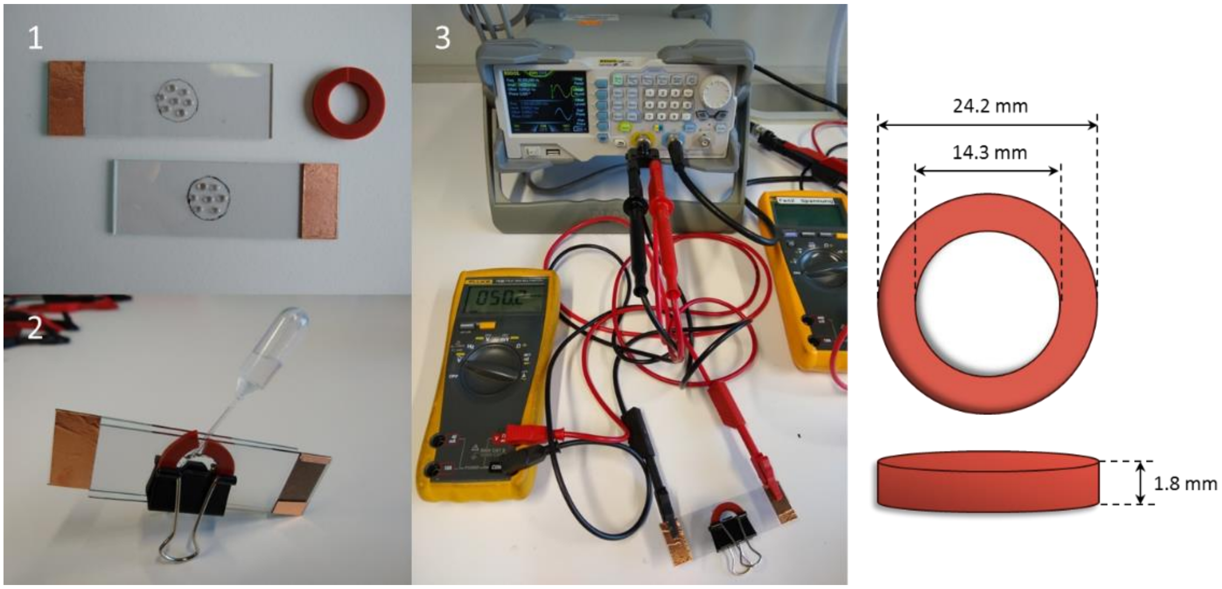

Figure S8. Setup for fusion/electroformation. Left: Preparation of proteoGUVs via fusion/electroformation. 1) Seven 2 µl droplets of proteoLUVs (5 mg ml^{-1}) deposited on ITO-coated glass slides, followed by dehydration for 40 min at room temperature. 2) Filling of the electroformation chamber with buffer. 3) Electroformation chamber connected to voltage generator. Right: Dimensions of electroformation spacer.

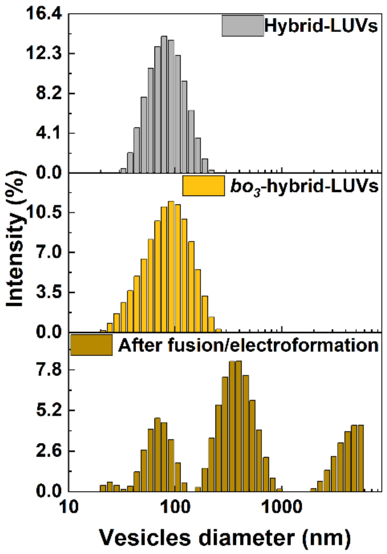

Figure S9. PDMS-*g*-PEO:PC (70:30, molar ratio) hybrids size distribution by intensity before and after reconstitution of *bo*$_3$ oxidase and after dehydration and electroformation, determined by DLS. Time of dehydration was only 30 min, which was insufficient and large portion of LUVs remained unfused.

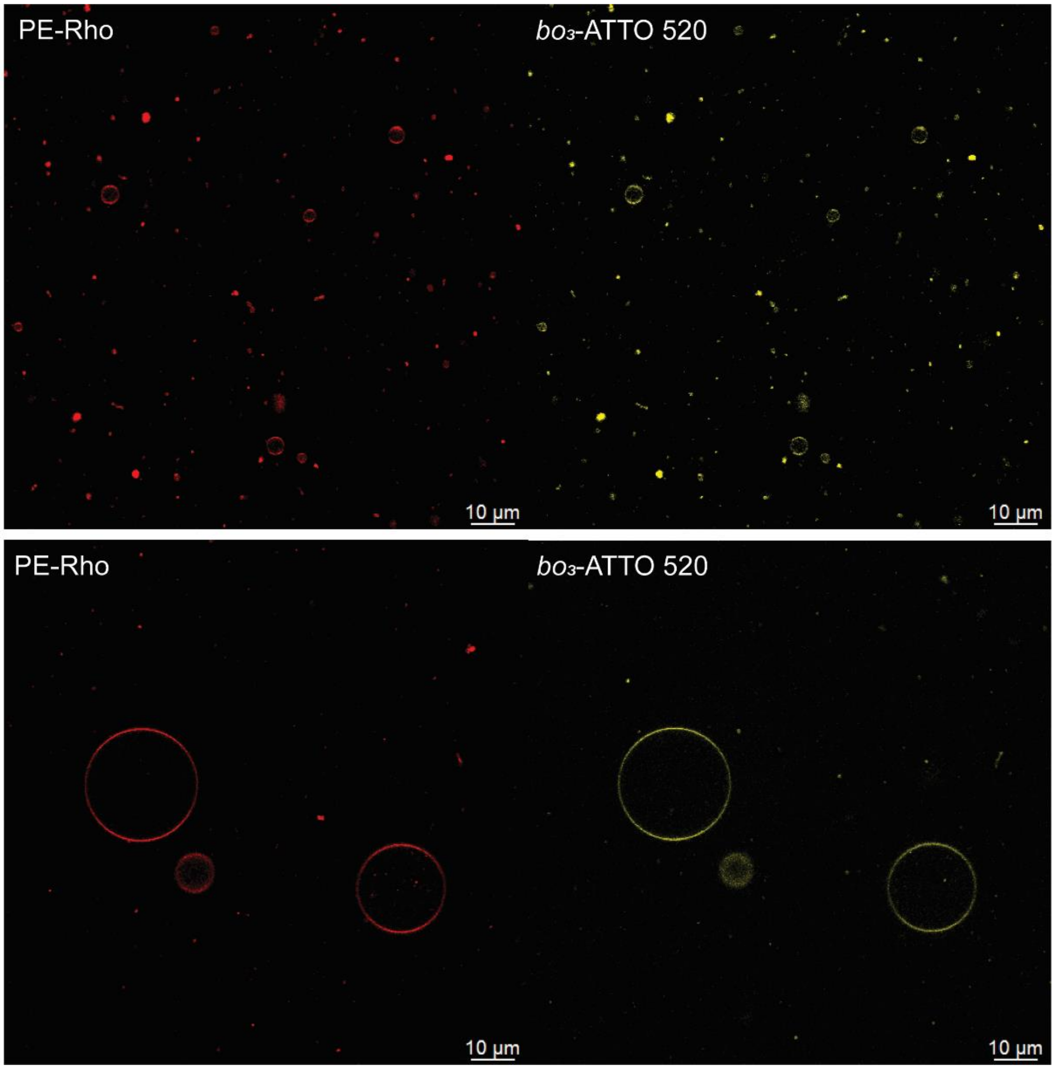

Figure S10. PDMS-*g*-PEO:PC GUVs with reconstituted *bo*$_3$ oxidase-ATTO 520 (yellow), prepared by rehydrating fused LUVs film under two different electroformation protocols: 500 Hz, 1.1 V, 3 h (panel above) and 50 Hz, 50, 100, 200, 300, 500, 700, 900, 1100 mV every 6 min, 50 Hz, 1.1 V overnight, 50 Hz, 1.5 V, 30 min (panel below; final protocol). The membrane was labeled with 0.03 mol% PE-Rho (red).

Figure S11–S12: Fusion/electroformation approach I and II

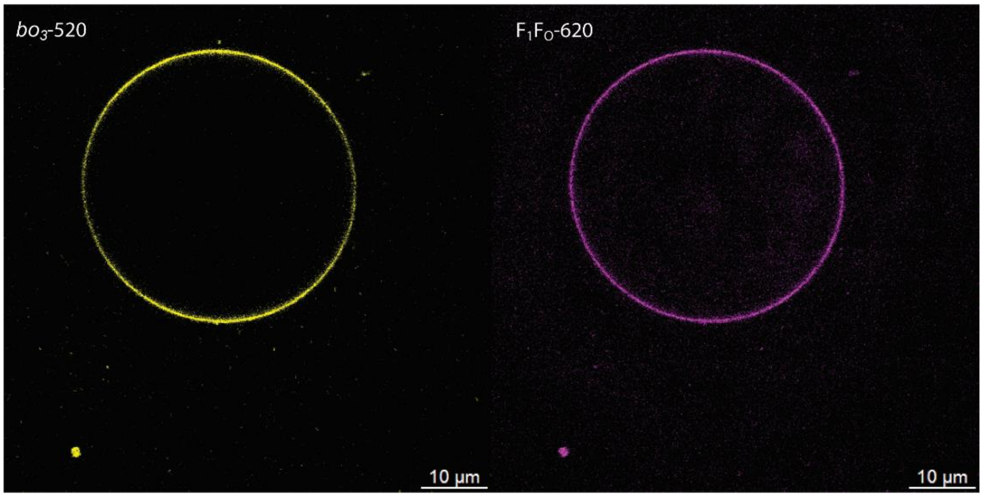

Figure S11. Hybrid GUVs with co-reconstituted *bo₃* oxidase-ATTO 514 (yellow) and F_1F_O-ATPase-ATTO 620 (magenta) formed by approach I.

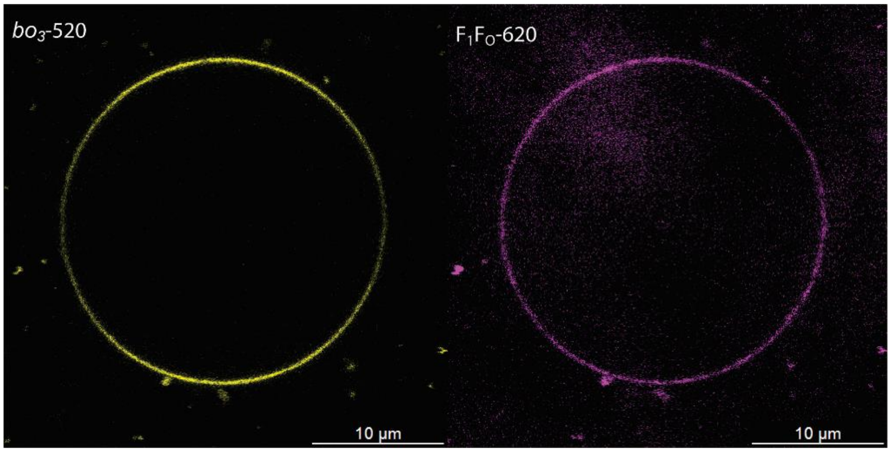

Figure S12. Hybrid GUVs with co-reconstituted *bo₃* oxidase-ATTO 514 (yellow) and F_1F_O-ATPase-ATTO 620 (magenta) formed by approach II.

Figure S13: Respiratory-driven ATP synthesis

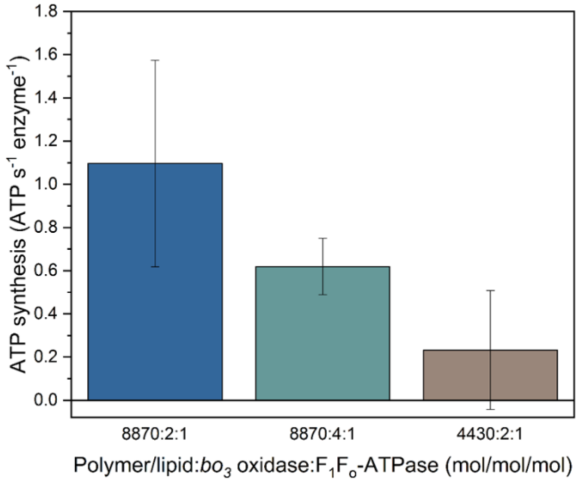

Figure S13. Respiratory-driven ATP synthesis by *bo3*-F_1F_O-hybrid-GUVs prepared by fusion/electroformation from *bo3*-LUVs and F_1F_O-LUVs at three different polymer/lipid-to-protein molar ratios.

Figure S14–S18: Confocal microscopy of bo_3-ATTO 425-F_1F_O-ATTO 620-hybrid-GUVs

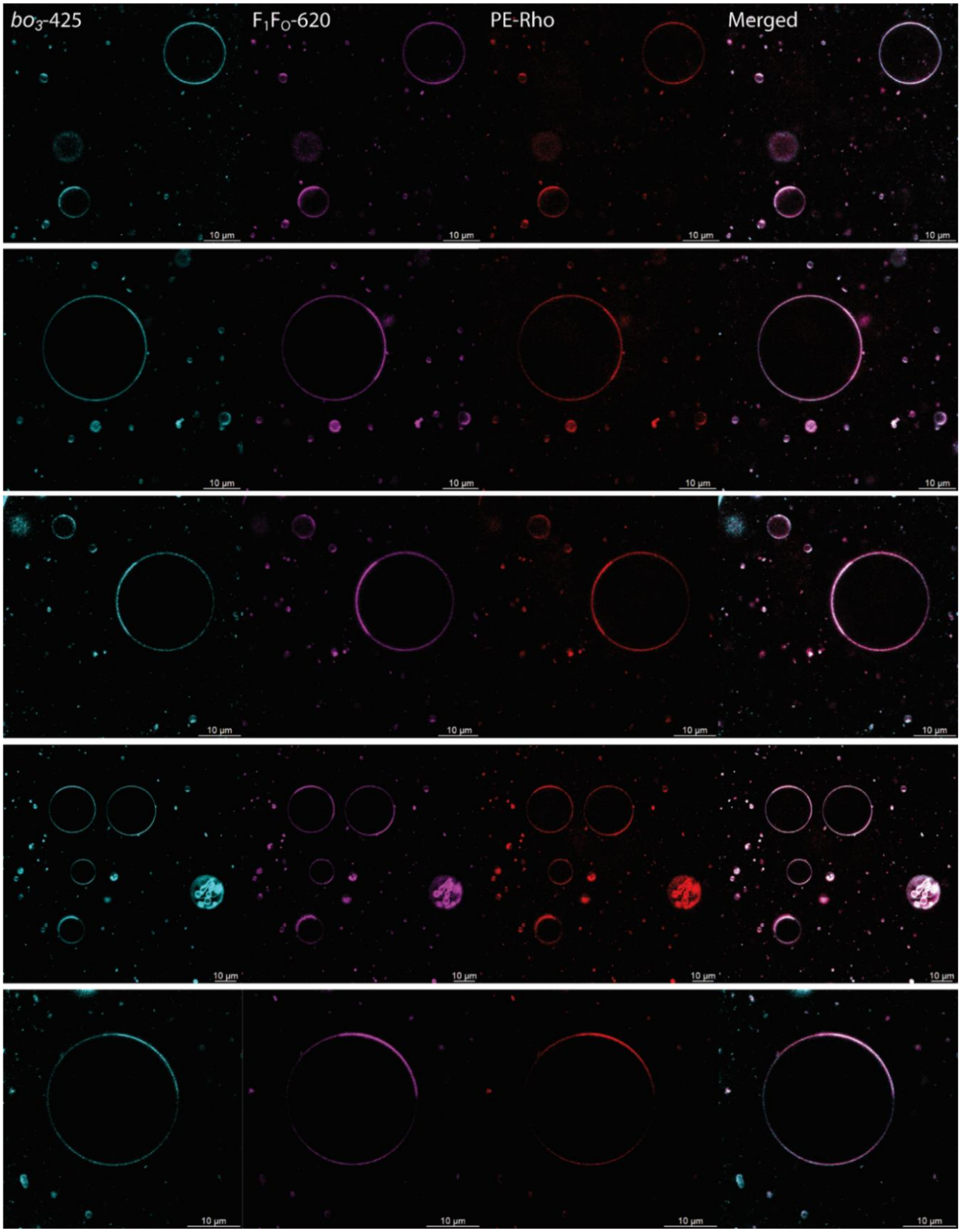

Figure S14. Phase separated hybrid GUVs with reconstituted bo_3 oxidase-ATTO 425 (cyan) and F_1F_O-ATPase-ATTO 620 (magenta) on day 4. Membrane was labeled with PE-Rho.

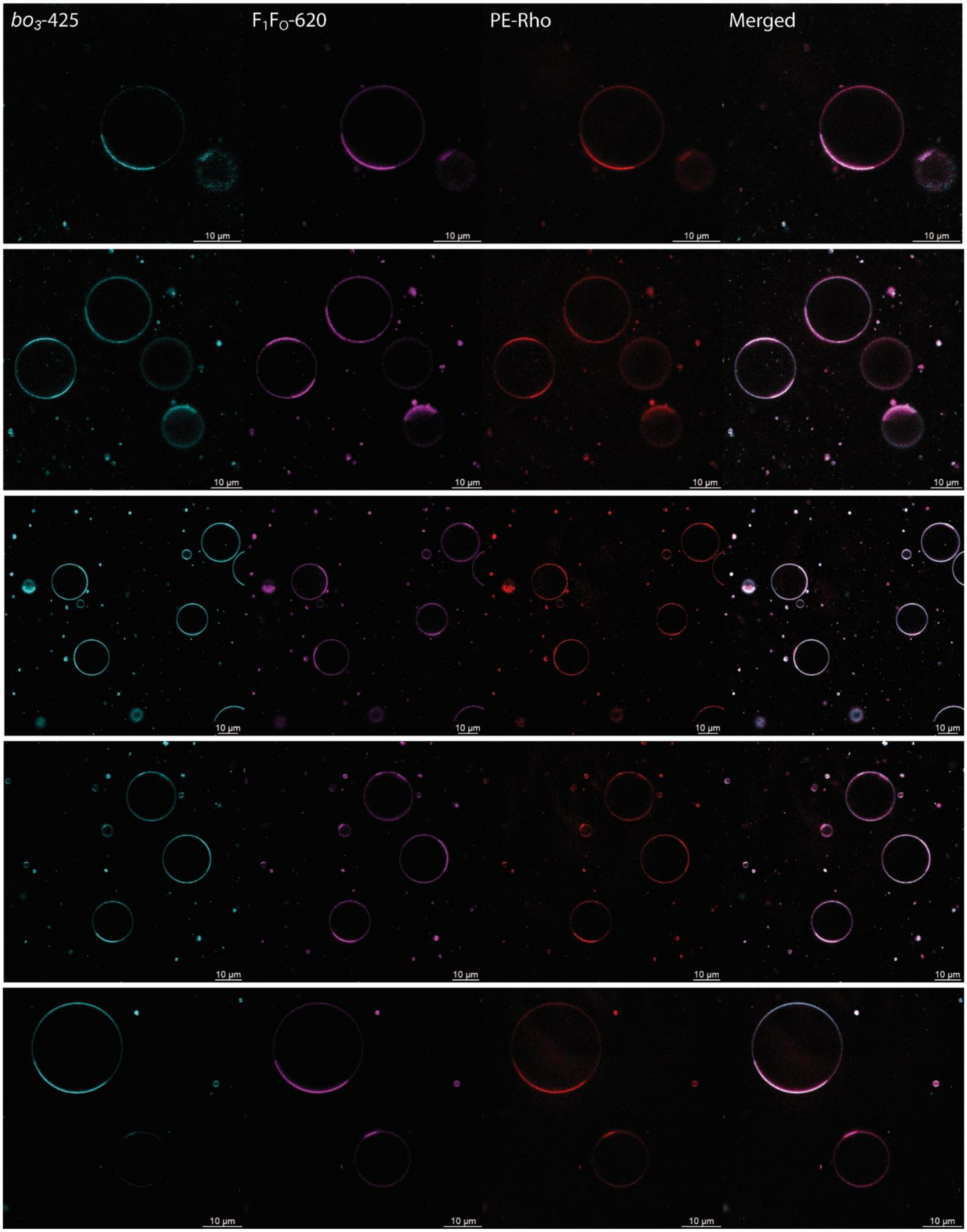

Figure S15. Phase separated hybrid GUVs with reconstituted bo_3 oxidase-ATTO 425 (cyan) and F_1F_O-ATPase-ATTO 620 (magenta) on day 4. Membrane was labeled with PE-Rho.

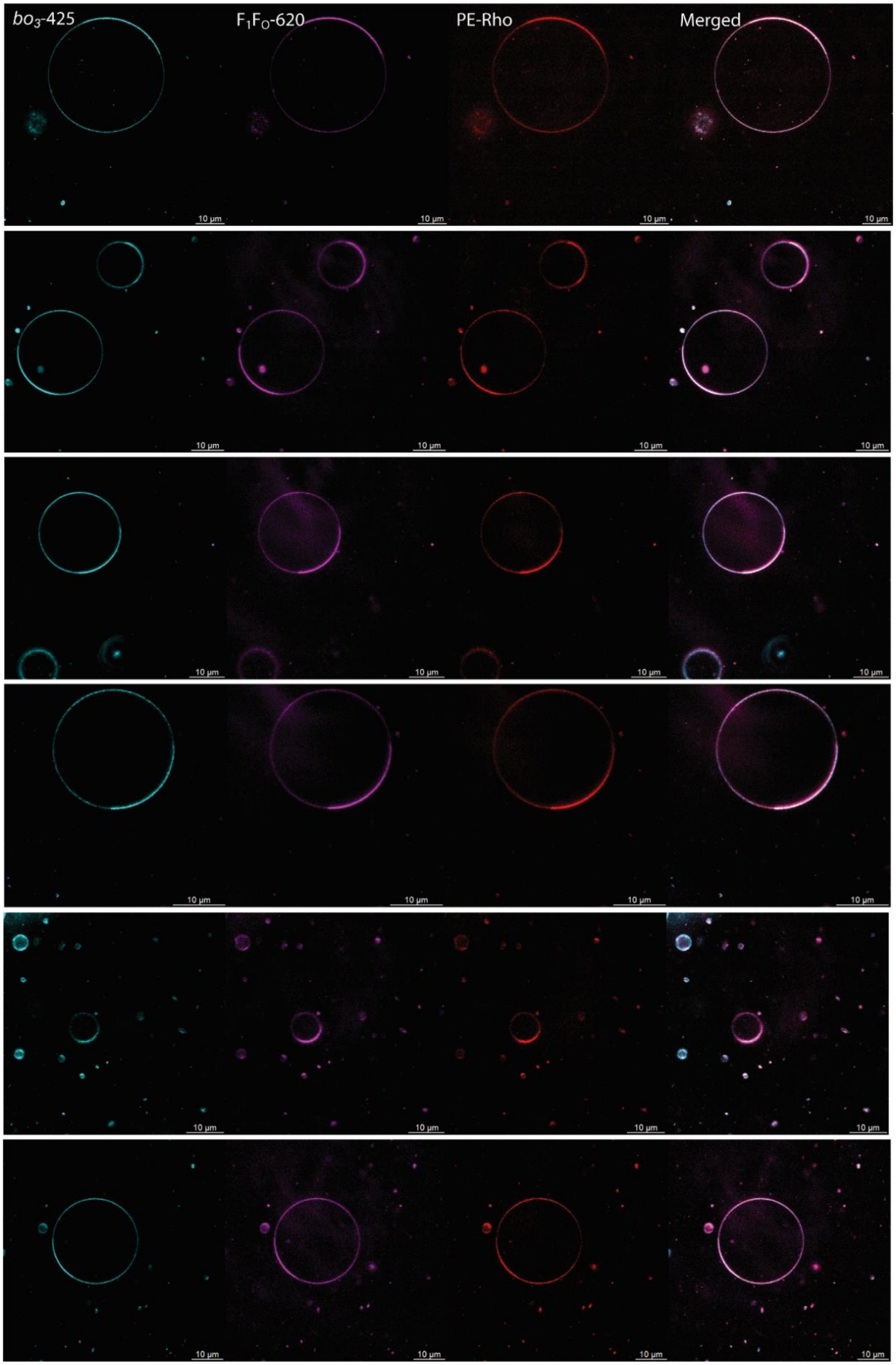

Figure S16. Phase separated hybrid GUVs with reconstituted *bo₃* oxidase-ATTO 425 (cyan) and F_1F_O-ATPase-ATTO 620 (magenta) on day 4. Membrane was labeled with PE-Rho (red).

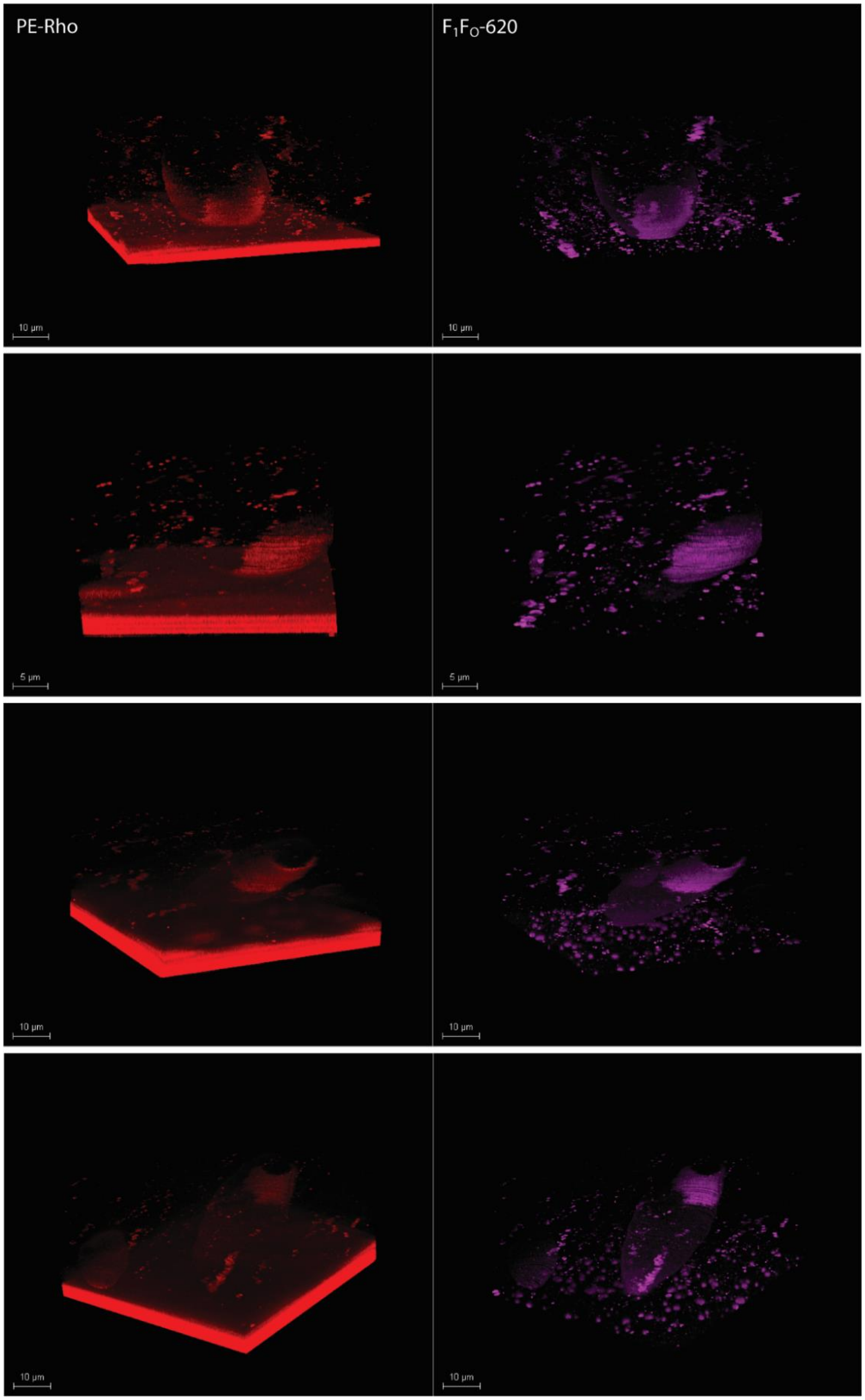

Figure S17. 3D of phase separated hybrid GUVs with reconstituted *bo₃* oxidase-ATTO 425 and F_1F_O-ATPase-ATTO 620 (magenta) on day 4. Membrane was labeled with PE-Rho (red). ATTO 425 channel is not shown due to high bleaching effect while taking Z-stacks.

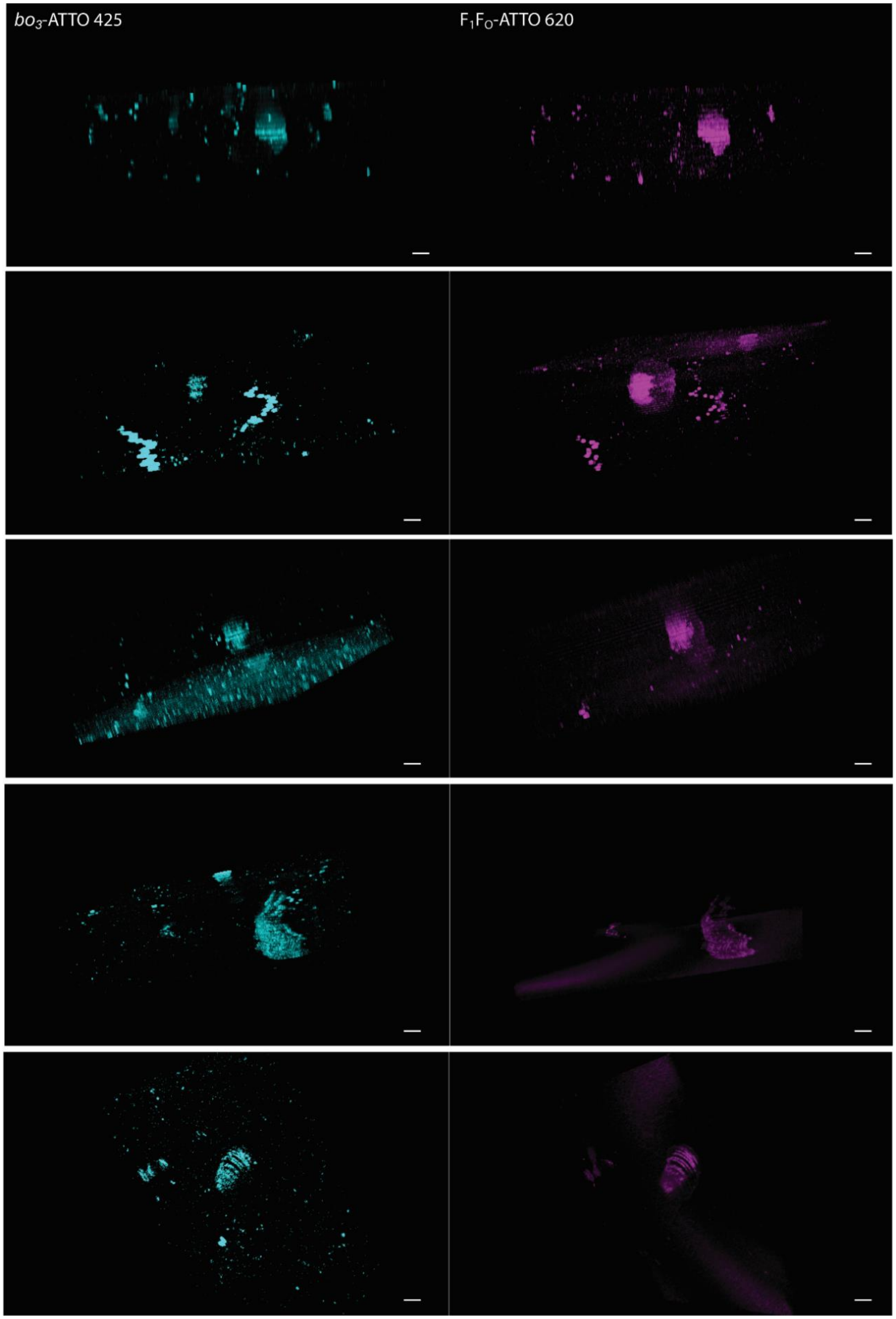

Figure S18. 3D of hybrid GUVs with reconstituted *bo₃* oxidase-ATTO 425 (cyan) and F_1F_O-ATTO 620 (magenta) on day 4. Scale bar: 5 μm.

Figure S19–S20: Confocal microscopy of *bo₃*-ATTO 520-F₁F₀-ATTO 620-hybrid-GUVs

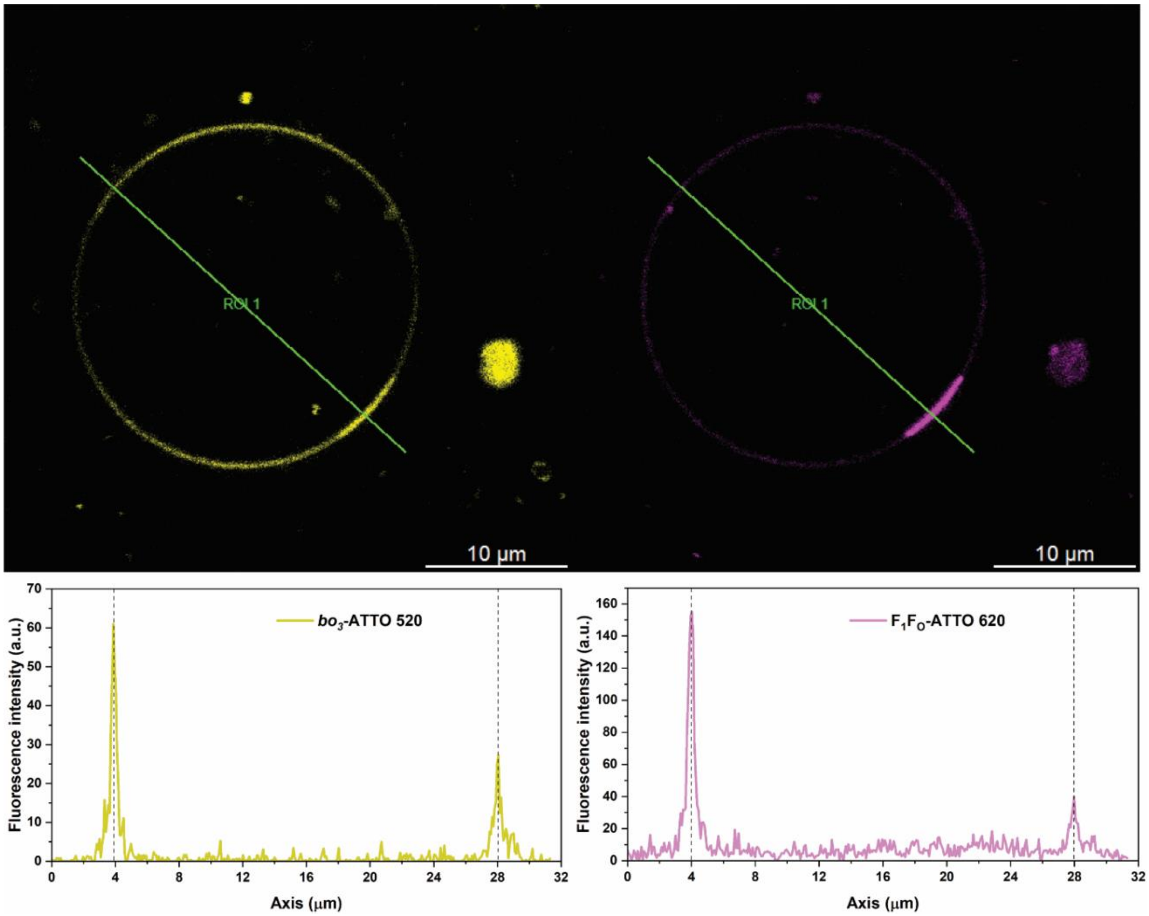

Figure S19. Line profile of hybrid GUV with co-for reconstituted *bo₃* oxidase-ATTO 520 (yellow) and F_1F_O-ATPase-ATTO 620 (magenta), formed by approach II.

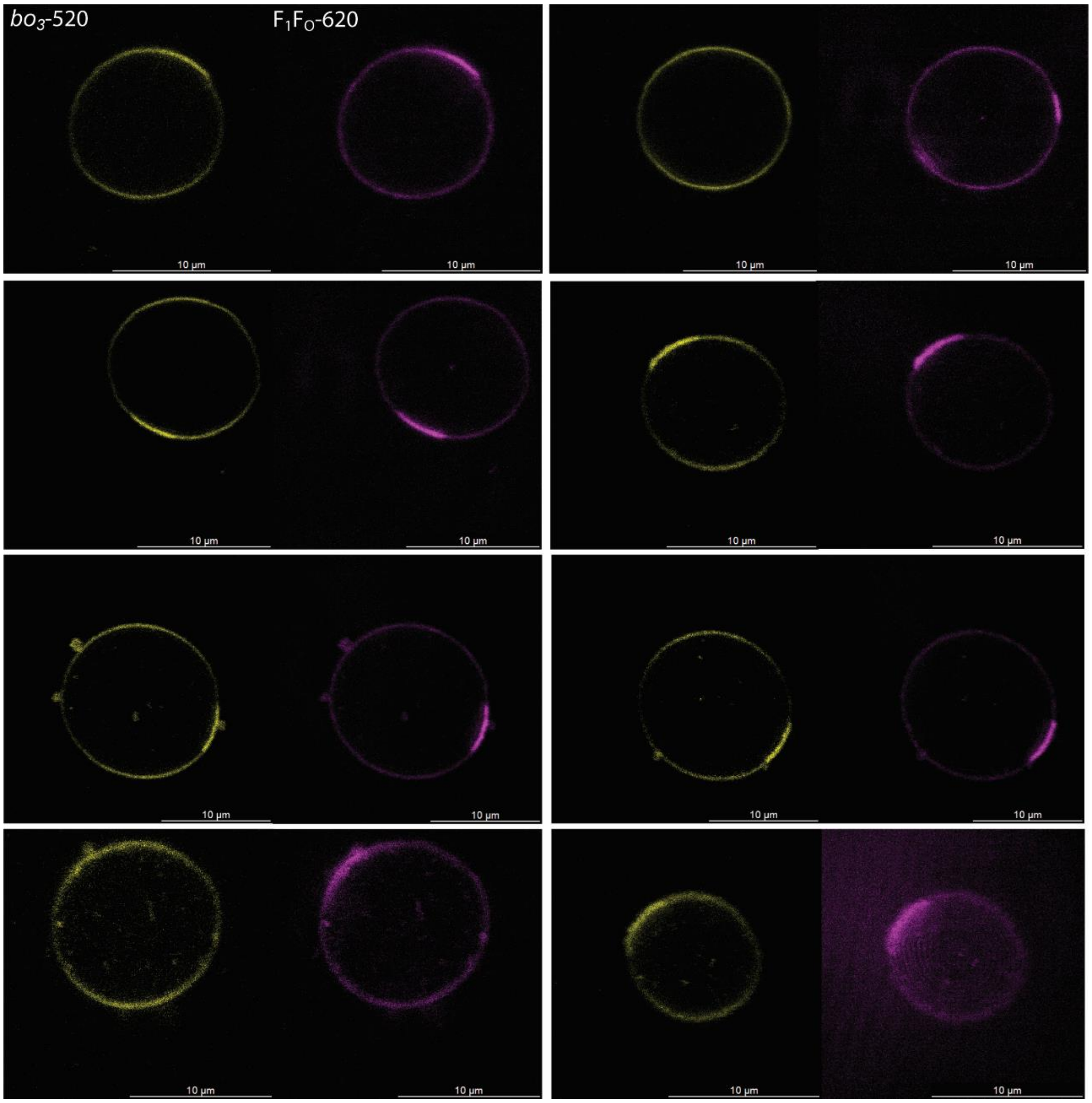

Figure S20. Phase separated hybrid GUVs with reconstituted bo_3 oxidase-ATTO 520 (yellow) and F_1F_O-ATPase-ATTO 620 (magenta) on day 4, formed by approach I.

Figure S21–S22: Confocal microscopy of *bo*$_3$-ATTO 514-F_1F_O-ATTO 620-hybrid-GUVs

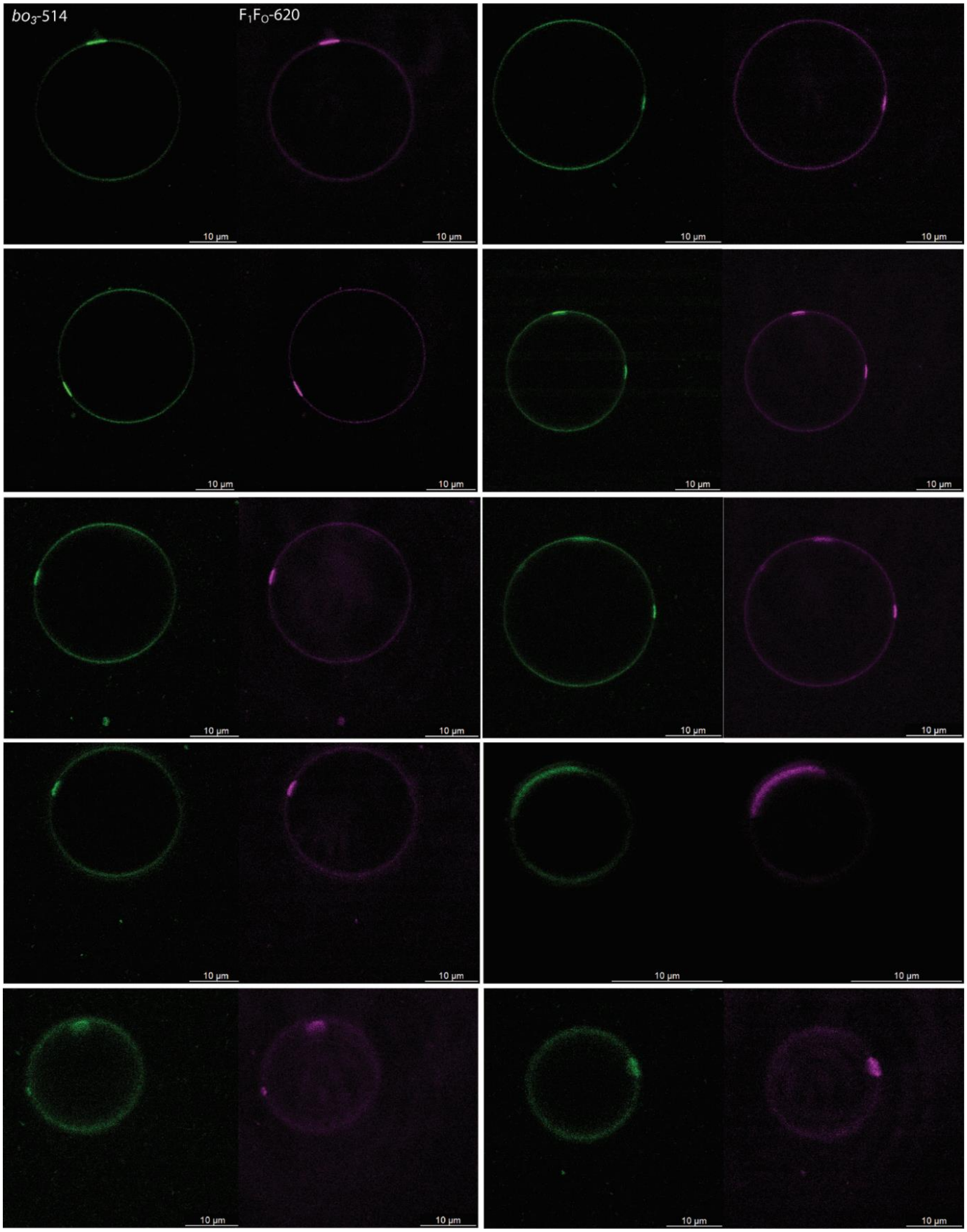

Figure S21. Phase separated hybrid GUVs with reconstituted *bo*$_3$-ATTO 514 (green) and F_1F_O-ATPase-ATTO 620 (magenta) on day 4.

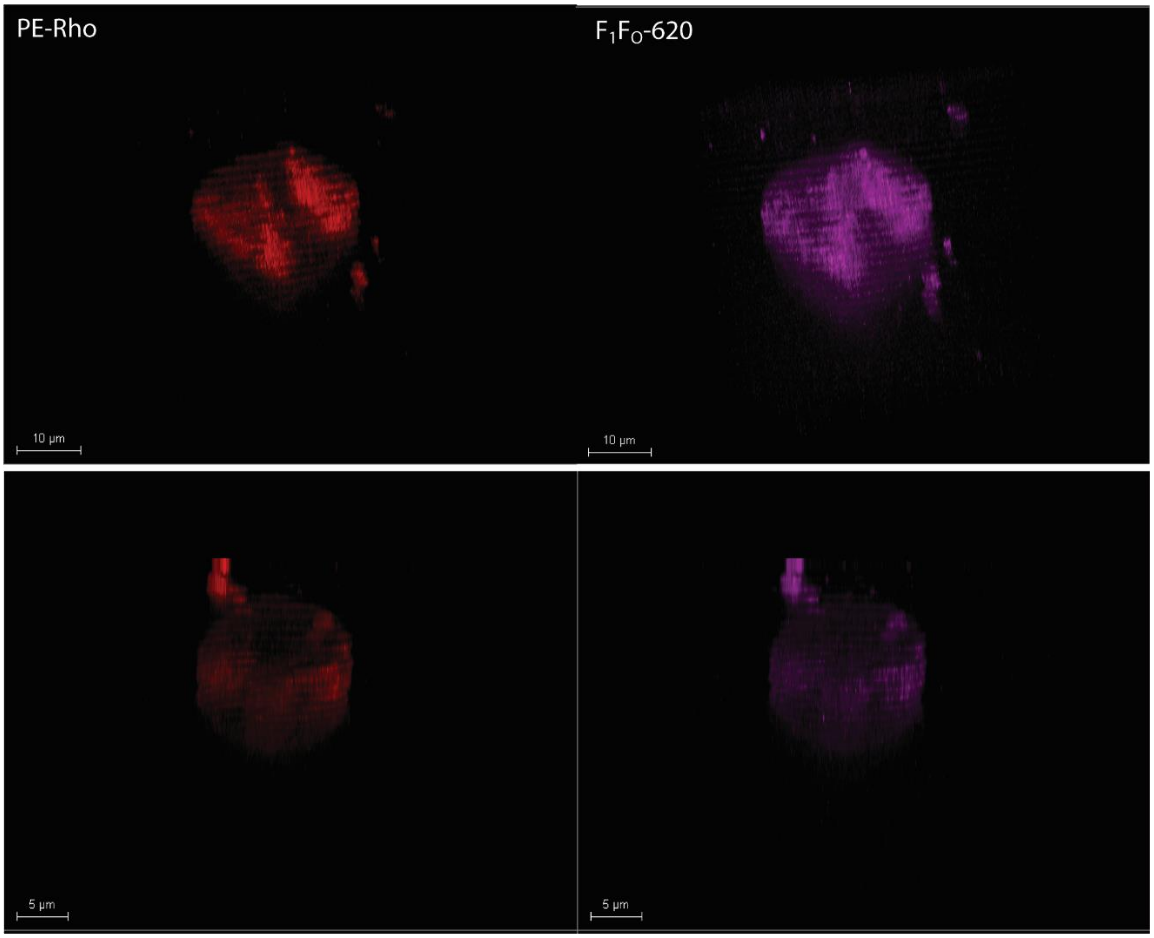

Figure S22. 3D of phase separated hybrid GUVs with reconstituted *bo₃* oxidase-ATTO 514 and F_1F_O-ATPase-ATTO 620 (magenta) on day 4. Membrane was labeled with PE-Rho (red).

Figure S23–S27: Confocal microscopy of *bo3*-ATTO 425/514/520-hybrid-GUVs

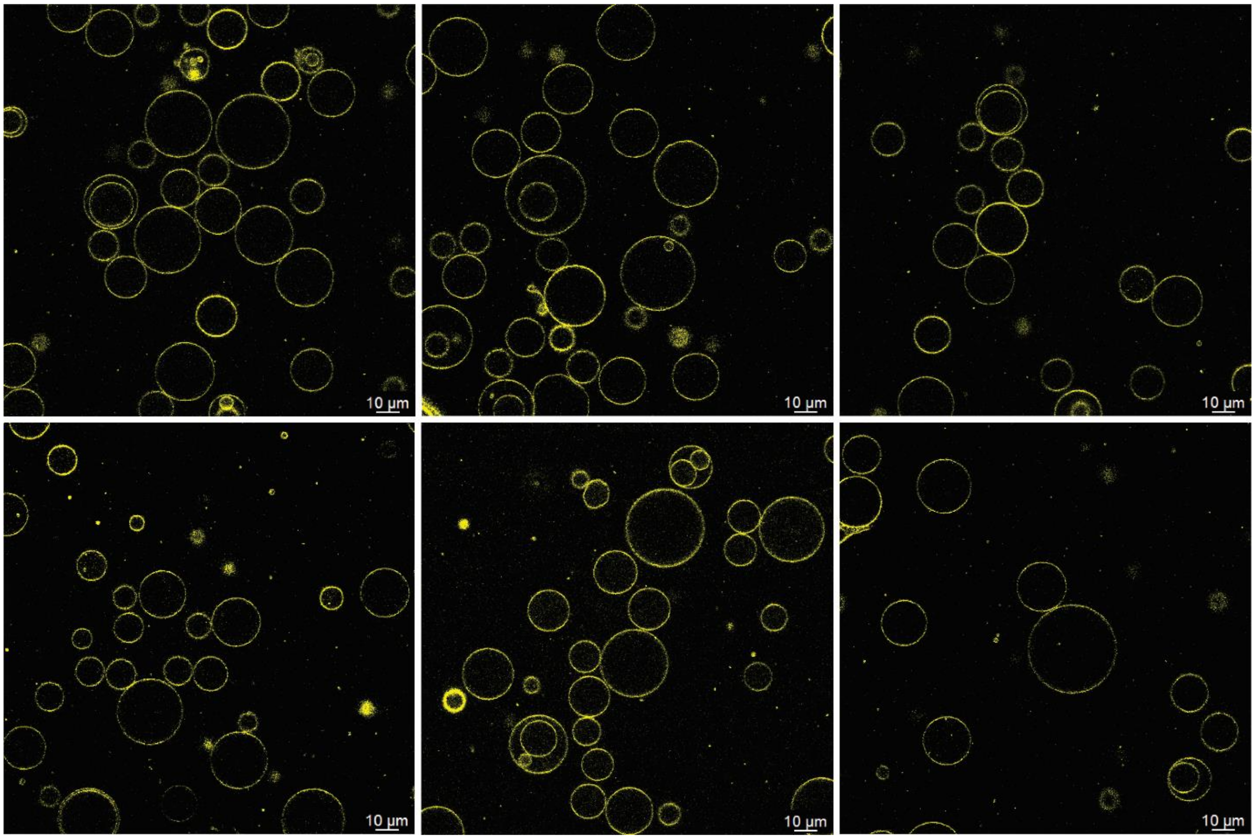

Figure S23. Hybrid GUVs with reconstituted *bo3* oxidase-ATTO 520 (yellow).

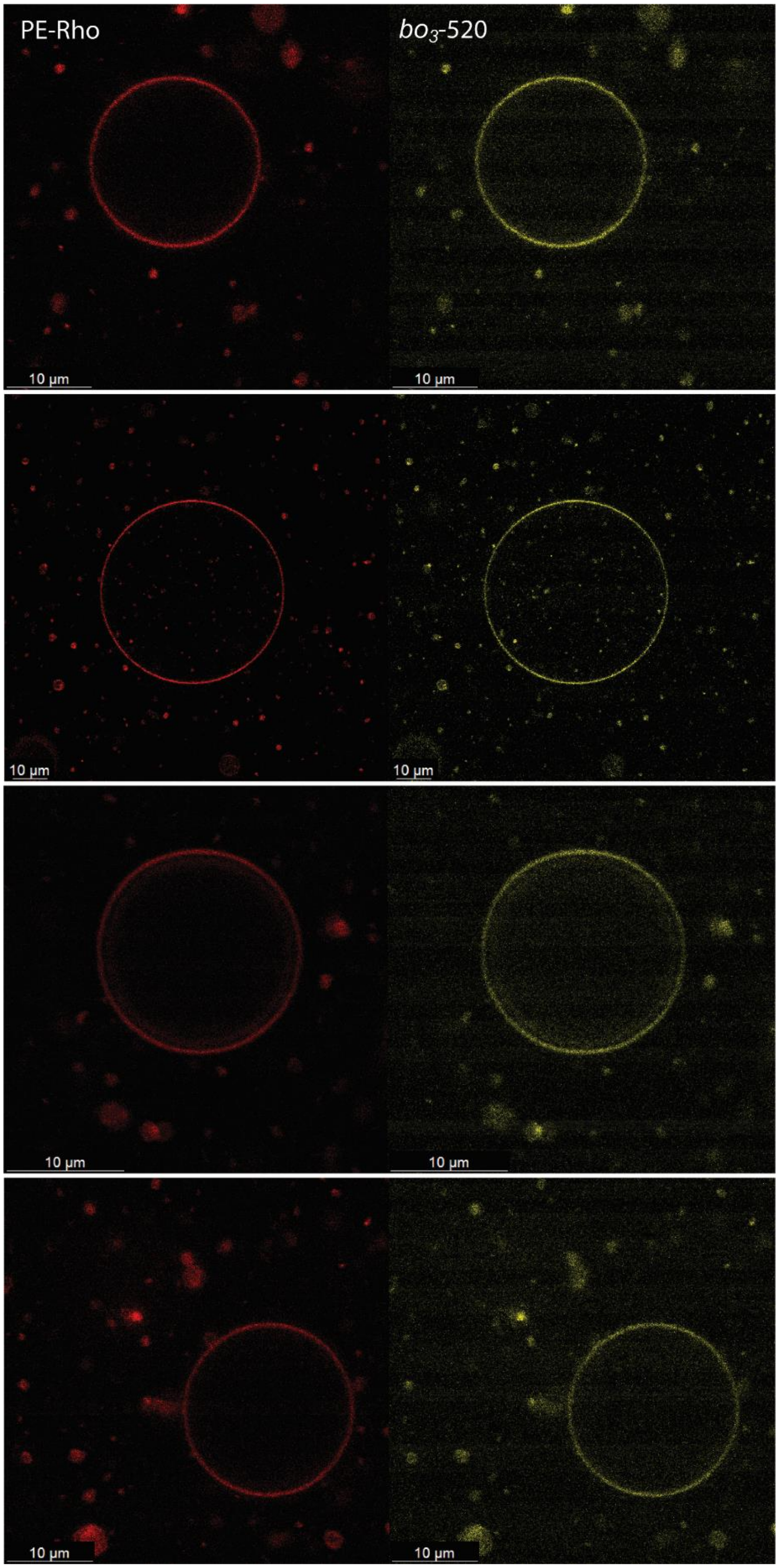

Figure S24. Hybrid GUVs with reconstituted bo_3 oxidase-ATTO 520 (yellow). Membrane was labeled with PE-Rho (red).

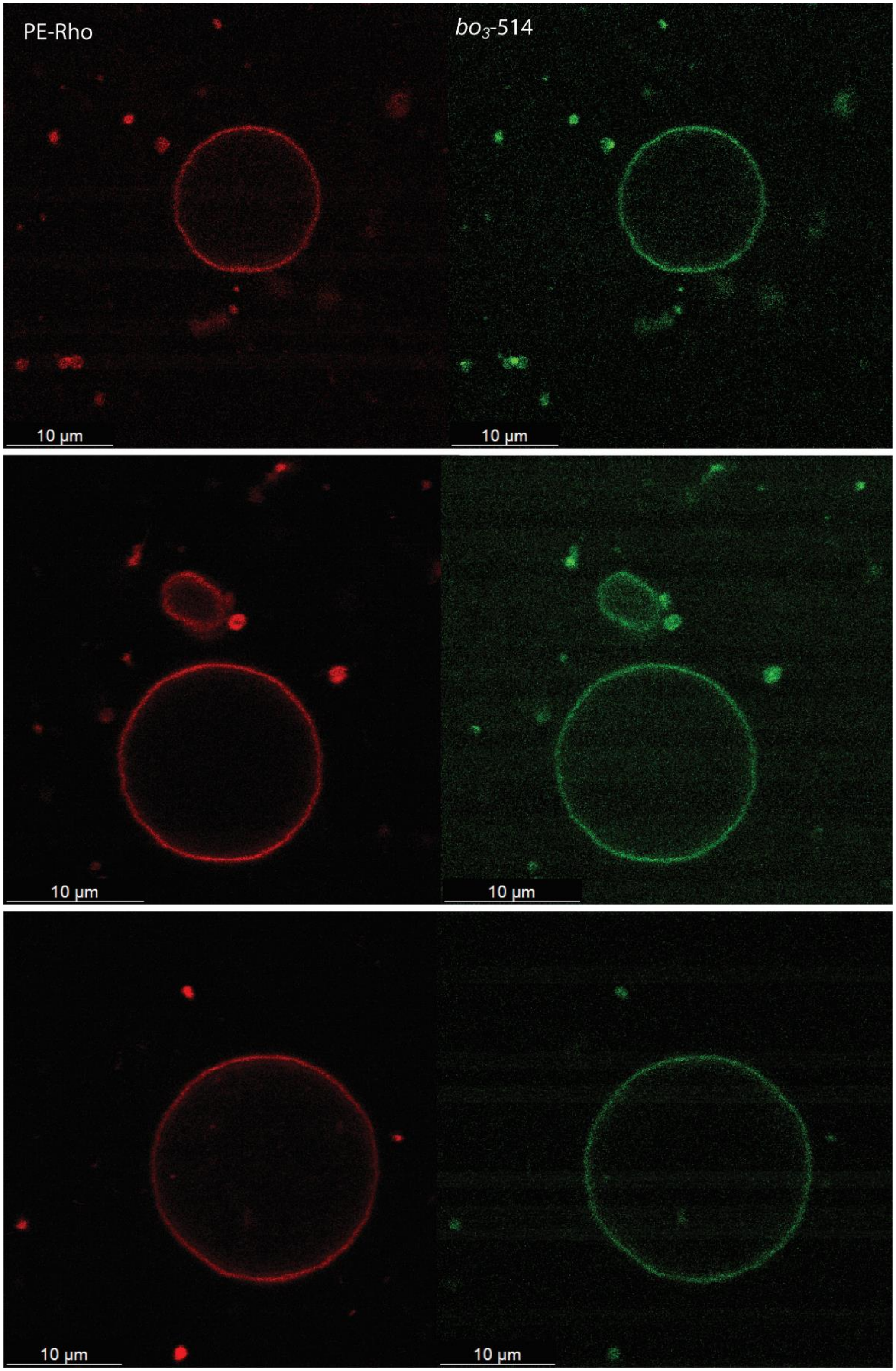

Figure S25. Hybrid GUVs with reconstituted bo_3 oxidase-ATTO 514 (green). Membrane was labeled with PE-Rho (red).

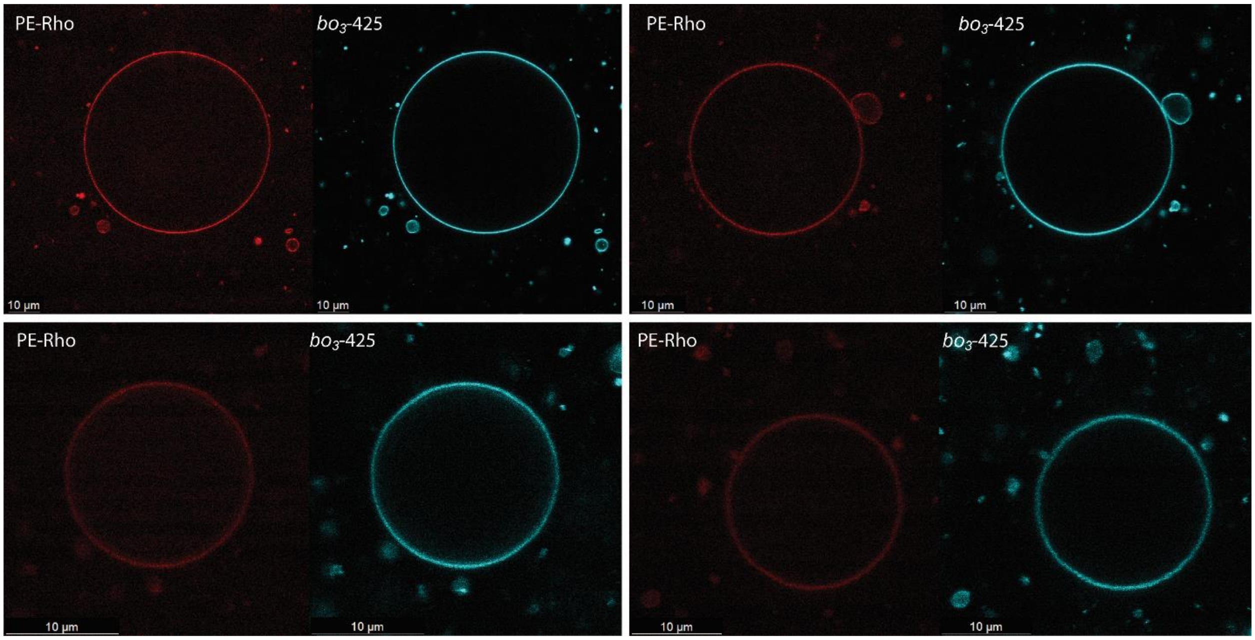

Figure S26. Hybrid GUVs with reconstituted *bo₃* oxidase-ATTO 425 (cyan) on day 1. Membrane was labeled with PE-Rho (red).

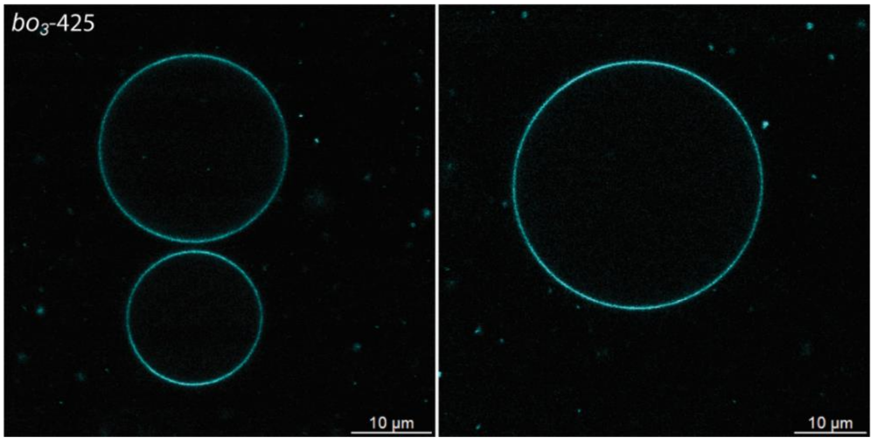

Figure S27. Hybrid GUVs with reconstituted *bo₃* oxidase-ATTO 425 (cyan) on day 4.

Figure S28–S30: Confocal microscopy of F_1F_O-ATTO 620-hybrid-GUVs

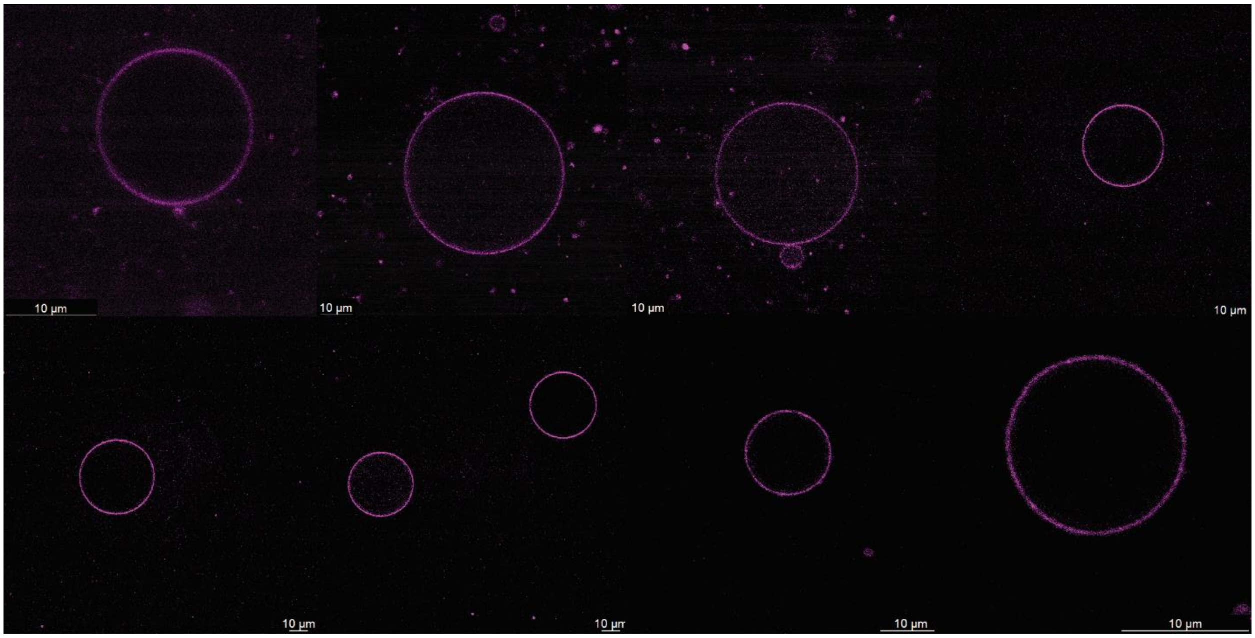

Figure S28. Hybrid GUV with reconstituted F_1F_O-ATPase-ATTO 620 (magenta) on day 1.

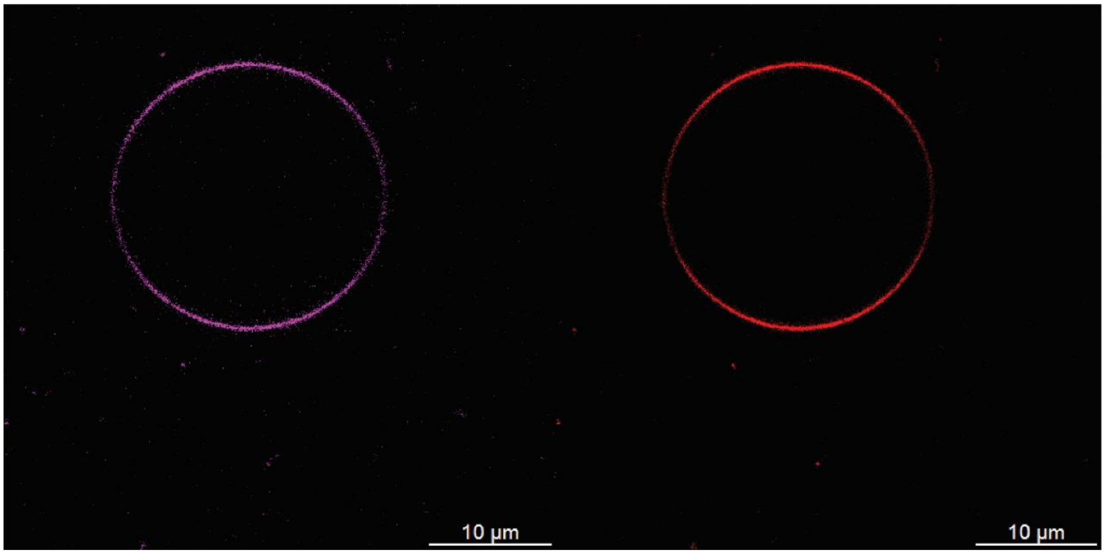

Figure S29. Hybrid GUV with reconstituted F_1F_O-ATPase-ATTO 620 (magenta) on day 1. Membrane was labeled with PE-Rho (red).

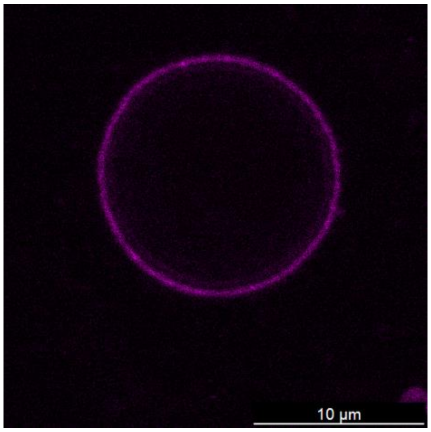

Figure S30. Hybrid GUV with reconstituted F_1F_O-ATPase-ATTO 620 (magenta) on day 4.

Figure S31: Confocal microscopy of *bo3*-ATTO 514-F1FO-ATTO 620-hybrid-GUVs on day 1

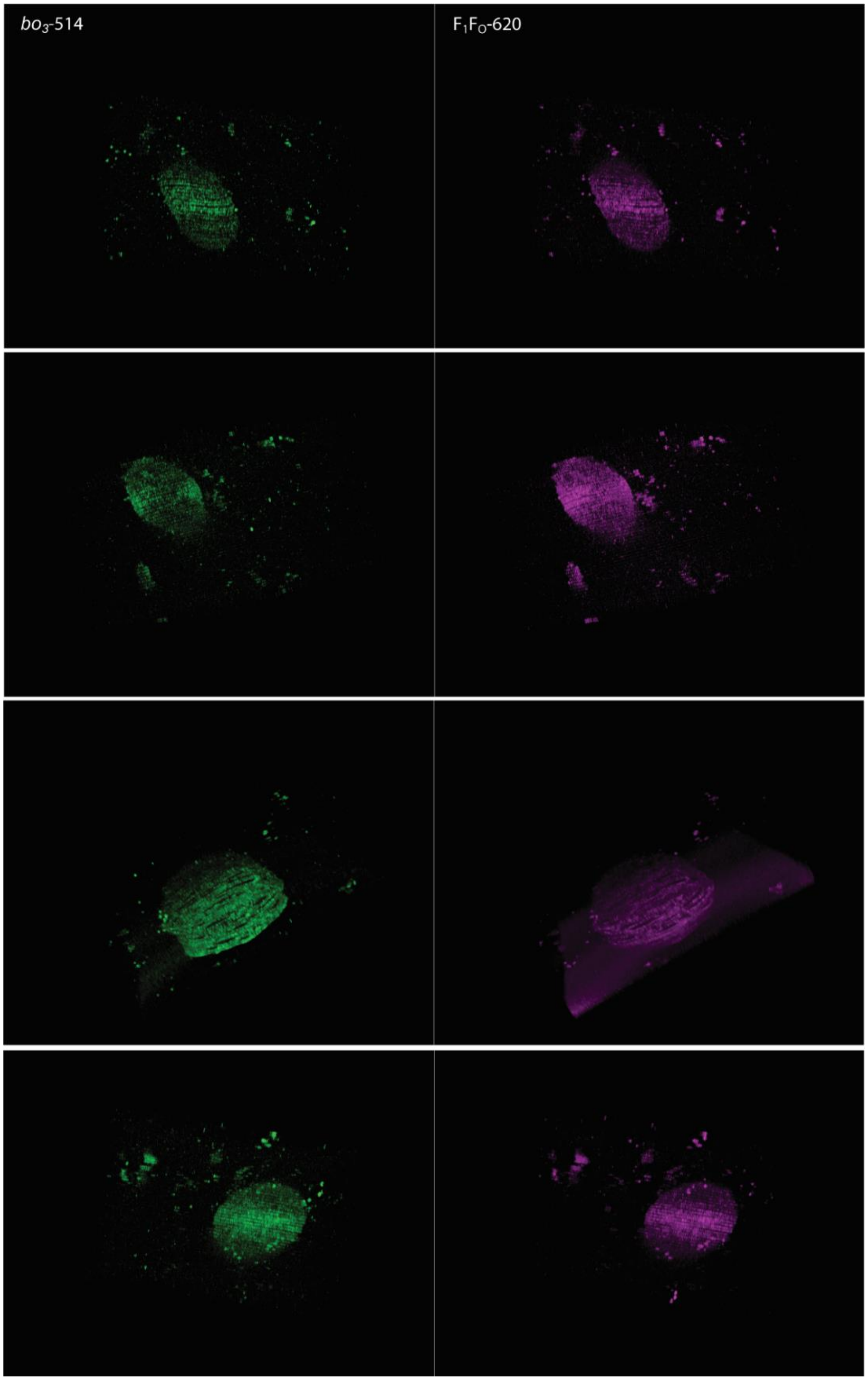

Figure S31. 3D of hybrid GUVs with reconstituted *bo3*-ATTO 514 (green) and F1FO-ATPase-ATTO 620 (magenta) on day 1.

Figure S32: Confocal microscopy of *bo₃*-F₁Fₒ-hybrid-GUVs

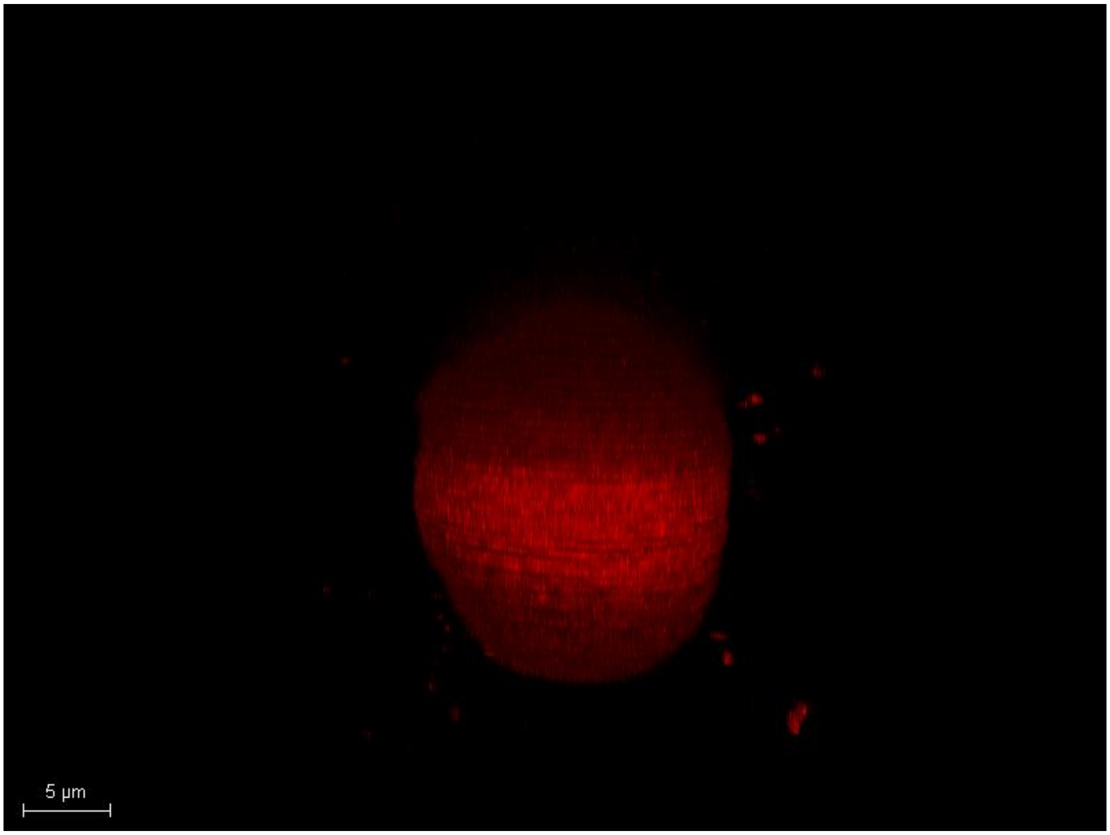

Figure S32. Typical hybrid GUV with co-reconstituted *bo_3* oxidase and F_1F_O-ATPase on day 4. All 68 evaluated *bo_3*-F_1F_O-GUVs had homogenous distribution of lipid dye (PE-Rho, red) in 2D, but higher dye intensity was observed in equatorial region after 3D analysis.

Figure S33: Confocal microscopy of protein partitioning in heterogeneous hybrid GUVs

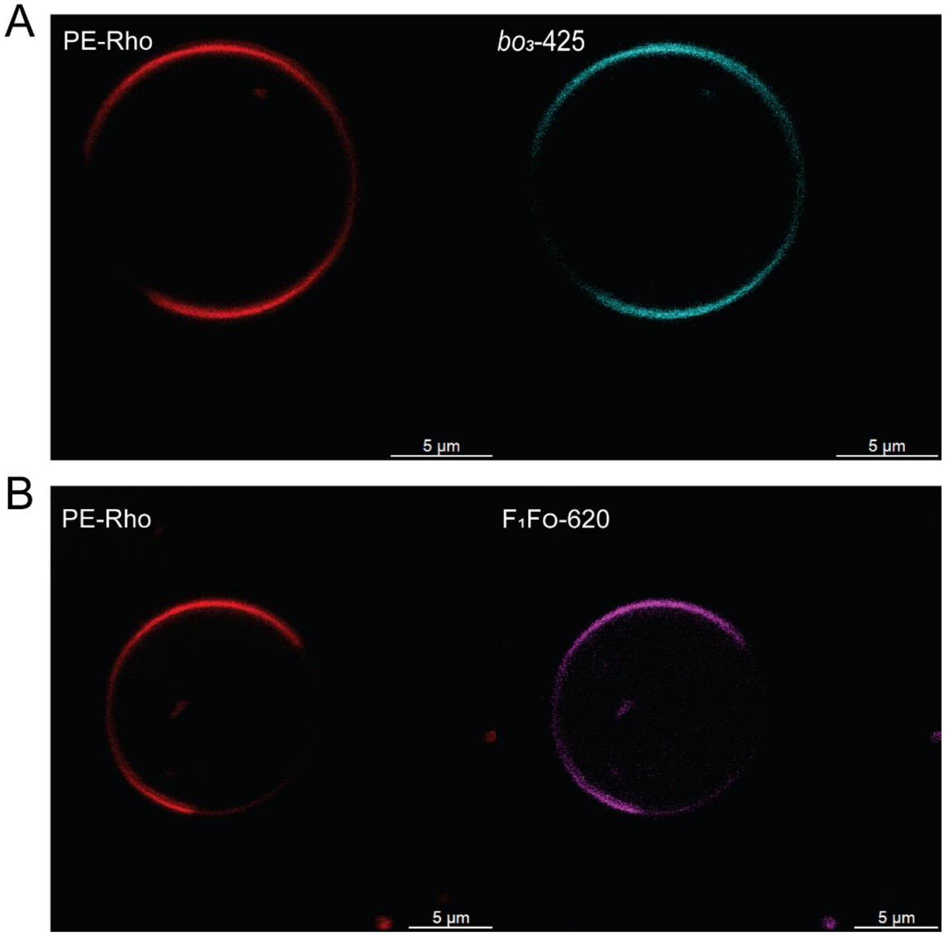

Figure S33. Insertion and partitioning of *bo3* oxidase-ATTO 425 (cyan) and F_1F_O-ATPase-ATTO 620 (magenta) in lipid domains (red) of heterogeneous hybrid GUV. A) *bo3*-425-hybrid-GUV, B) F_1F_O-620-hybrid-GUV.

Figure S34: Fluorescence intensity of proteins in heterogeneous and homogenous hybrid GUVs

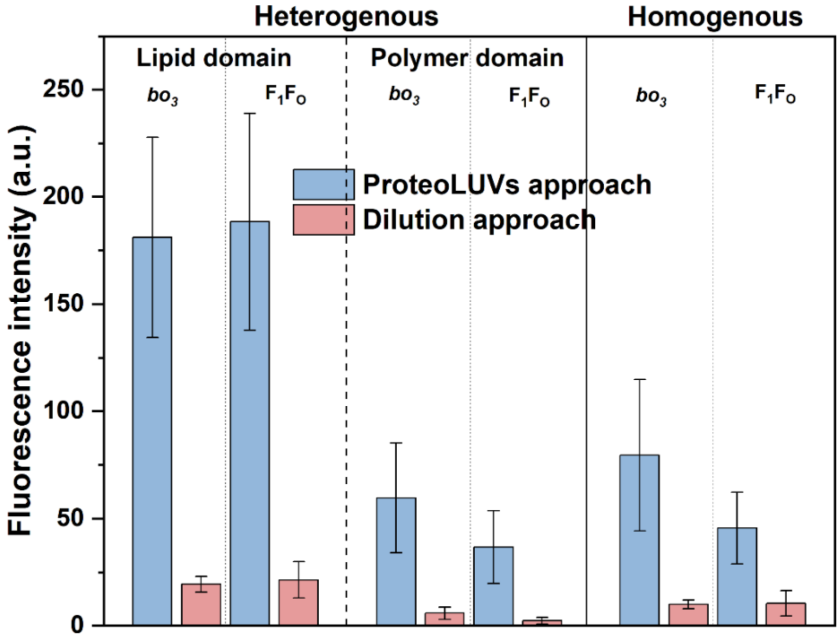

Figure S34. Fluorescence intensity of *bo₃* oxidase-ATTO 425 and F_1F_O-ATPase-ATTO 620 in heterogeneous and homogenous hybrid GUVs. ProteoGUVs were formed either from proteoLUVs via fusion/electroformation approach (blue) or dilution approach (red).

Video S1–S2

Video S1. Hybrid GUV with co-reconstituted bo_3 oxidase labeled with ATTO 425 (cyan) and F_1F_O-ATPase-ATTO 620 (magenta) on day 4.

Video S2. Hybrid GUV with co-reconstituted bo_3 oxidase labeled with ATTO 520 (yellow) and F_1F_O-ATPase-ATTO 620 (magenta) on day 4.

References

1. Schägger, H. and von Jagow, H., *Tricine-sodium dodecyl sulfate-polyacrylamide gel electrophoresis for the separation of proteins in the range from 1 to 100 kDa.* Anal. Biochem., 1987. **166**(2): p. 368-79.
2. Ishmukhametov, R.R., et al., *Ultrafast purification and reconstitution of His-tagged cysteine-less Escherichia coli F_1F_o ATP synthase.* Biochim. Biophys. Acta Bioenerg., 2005. **1706**(1-2): p. 110-6.
3. Rumbley, J.N., et al., *One-step purification of histidine-tagged cytochrome bo_3 from Escherichia coli and demonstration that associated quinone is not required for the structural integrity of the oxidase.* Biochim. Biophys. Acta, 1997. **1340**(1): p. 131-42.
4. Seneviratne, R., et al., *A reconstitution method for integral membrane proteins in hybrid lipid-polymer vesicles for enhanced functional durability.* Methods, 2018. **147**: p. 142-149.
5. Marušič, N., et al., *Constructing artificial respiratory chain in polymer compartments: Insights into the interplay between bo_3 oxidase and the membrane.* Proc. Natl. Acad. Sci. U.S.A., 2020. **117**(26): p. 15006–15017.
6. Rigaud, J.L., et al, *Reconstitution of membrane proteins into liposomes: application to energy-transducing membrane proteins.* Biochim. Biophys. Acta, 1995. **1231**(3): p. 223-46.
7. Otrin, L., et al., *Toward Artificial Mitochondrion: Mimicking Oxidative Phosphorylation in Polymer and Hybrid Membranes.* Nano Lett., 2017. **17**(11): p. 6816-6821.
8. Chung, D.-w. and J.C. Lim, *Study on the effect of structure of polydimethylsiloxane grafted with polyethyleneoxide on surface activities.* Colloids Surf., A, 2009. **336**(1): p. 35-40.
9. Girard, P., et al., *A new method for the reconstitution of membrane proteins into giant unilamellar vesicles.* Biophys. J., 2004. **87**(1): p. 419-29.
